# Characterization and Biosynthetic Regulation of Isoflavone Genistein in Deep-Sea Actinomycetes *Microbacterium* sp. B1075

**DOI:** 10.3390/md22060276

**Published:** 2024-06-13

**Authors:** Xin Li, Yukun Cui, Weichao Wu, Zhizhen Zhang, Jiasong Fang, Xi Yu, Junwei Cao

**Affiliations:** 1 College of Oceanography and Ecological Science, Shanghai Ocean University, Shanghai 201306, China; lixin980808@163.com (X.L.); 18638405798@sina.cn (Y.C.); wcwu@shou.edu.cn (W.W.); jsfang@shou.edu.cn (J.F.); 2 Ocean College, Zhoushan Campus, Zhejiang University, Zhoushan 316021, China; zzhang88@zju.edu.cn

**Keywords:** deep-sea actinomycetes, biological activity, flavonoid compounds, biosynthetic genes

## Abstract

Deep-sea environments, as relatively unexplored extremes within the Earth’s biosphere, exhibit notable distinctions from terrestrial habitats. To thrive in these extreme conditions, deep-sea actinomycetes have evolved unique biochemical metabolisms and physiological capabilities to ensure their survival in this niche. In this study, five actinomycetes strains were isolated and identified from the Mariana Trench via the culture-dependent method and 16S rRNA sequencing approach. The antimicrobial activity of *Microbacterium* sp. B1075 was found to be the most potent, and therefore, it was selected as the target strain. Molecular networking analysis via the Global Natural Products Social Molecular Networking (GNPS) platform identified 25 flavonoid compounds as flavonoid secondary metabolites. Among these, genistein was purified and identified as a bioactive compound with significant antibacterial activity. The complete synthesis pathway for genistein was proposed within strain B1075 based on whole-genome sequencing data, with the key gene being *CHS* (encoding chalcone synthase). The expression of the gene *CHS* was significantly regulated by high hydrostatic pressure, with a consequent impact on the production of flavonoid compounds in strain B1075, revealing the relationship between actinomycetes’ synthesis of flavonoid-like secondary metabolites and their adaptation to high-pressure environments at the molecular level. These results not only expand our understanding of deep-sea microorganisms but also hold promise for providing valuable insights into the development of novel pharmaceuticals in the field of biopharmaceuticals.

## 1. Introduction

The deep sea, constituting approximately seventy-five percent of the ocean’s total volume, manifests extreme physicochemical conditions relative to terrestrial environments [1]. These conditions include high salinity, elevated hydrostatic pressure, low temperatures, oligotrophy, and an absence of light. Despite these extreme environmental conditions, the deep sea still harbors vast and diverse ecosystems, nurturing metabolically active and diversified microbial communities [2].

Over the past four decades, marine actinomycetes have become a major source of natural products in the ocean, with significant potential for producing bioactive compounds [3]. The exposure of marine actinomycetes to extreme oceanic environments and distinct microbial competitive landscapes has engendered a diverse range of genetic functions and the uniqueness of ecological functionalities [4]. In response to these environments, certain actinomycetes have evolved distinctive metabolic capabilities, with a high probability of generating pharmacologically compounds not observed among terrestrial actinomycetes [5]. Consequently, there is a growing trend of researching rare actinomycetes from various environments to uncover their potential pharmacological benefits [6,7,8]. It is worth noting that *Microbacterium* is the most commonly identified genus among deep-sea actinomycetes [5,9]. While research on *Microbacterium* species typically focuses on activities like environmental pollutant degradation, there is limited research regarding their potential in drug development [10,11]. The assessment of antimicrobial activity, a pivotal criterion for assessing the utility of actinomycetes, remains a guiding principle in the classical approach to investigating the secondary metabolites of actinomycetes.

Flavonoids are a class of physiologically and ecologically active secondary metabolites derived from the phenylpropanoid metabolic pathway. Genistein belongs to the flavonoid group and is specifically classified as an isoflavone [12]. It serves as a plant-derived phytoestrogen and is predominantly found in legumes and occasionally in other plants. Genistein is considered a promising lead compound for drug development [13] and has been reported to have numerous beneficial effects on health, such as preventing osteoporosis, reducing cardiovascular risks, alleviating postmenopausal symptoms, and possessing anti-cancer properties. The biosynthesis of genistein has also been reported in the culturing of bacteria and fungi. In bacteria, heterologous biosynthesis typically favors the use of tyrosine ammonia-lyase (TAL) over phenylalanine ammonia-lyase (PAL) [14]. This preference arises because the utilization of tyrosine as the starting substrate eliminates the need for the activity of cinnamate 4-hydroxylase (4CH), as the amino acid is already hydroxylated at the required position. Subsequently, in the ensuing enzymatic reaction, chalcone synthase (CHS) condenses one molecule of 4-coumaroyl-CoA with three molecules of malonyl-CoA to yield the chalcone of genistein, which represents the fundamental carbon scaffold of over 9000 known flavonoid compounds in nature [15]. In most microbial biosynthesis processes, genes responsible for the synthesis of the plant dye naringenin are often transferred to engineering microorganisms such as *Escherichia coli* and *Saccharomyces cerevisiae* [16]. However, natural metabolic pathways warrant further exploration due to inherent limitations in biological synthesis [17]. Synthesizing genistein from natural secondary metabolites may represent a novel metabolic pathway, potentially offering new insights and methods for the synthesis of isoflavone compounds. This approach holds the promise of enhancing the practical applications of such substances in real-life scenarios.

Actinomycetes, particularly Streptomyces species, are the main producers of flavonoid compounds in microorganisms. This is due to the presence of genes encoding key enzymes that catalyze the synthesis of chalcone and flavonoid compounds, such as the gene *CHS* [15]. *CHS*, isolated as the first flavonoid biosynthetic gene from parsley, holds significant importance in the synthesis pathway of flavonoids [18]. The chalcone synthase catalyzes the formation of a tetraketide intermediate, utilizing *p*-coumaroyl-CoA and malonyl-CoA to cyclize it into chalcone [12]. Since chalcone serves as a crucial substrate in the biosynthesis of flavonoids, the expression level of the gene *CHS* plays a pivotal role in the accumulation of flavonoids within the organism. At present, research on flavonoid secondary metabolites in actinomycetes mainly focuses on the Streptomyces genus, with rare instances of flavonoid compounds isolated from other less common actinomycete genera, not to mention deep-sea actinomycetes. Furthermore, there is a lack of comprehensive analysis of the molecular mechanisms of the biosynthetic pathways of flavonoid compounds with environmental regulation.

In this study, we screened deep-sea *Microbacterium* strains for antibacterial activity, selecting *Microbacterium* sp. B1075 as the target strain. The Global Natural Products Social Molecular Networking (GNPS) molecular networking approach was employed to identify secondary metabolites in strain B1075, with isoflavone being the most prominent. Purification and structural elucidation were then used to analyze the bioactive metabolites. The putative biosynthetic pathway involved in flavonoid biosynthesis was analyzed via whole-genome sequencing. Furthermore, we examined the alterations in the *CHS* expression and metabolite production, particularly flavonoids, under diverse high-pressure conditions. This research establishes a foundation for investigating bioactive secondary metabolites in deep-sea actinomycetes and elucidating isoflavonoid metabolic pathways in microorganisms.

## 2. Results

### 2.1. Isolation of Actinomycetes from Deep-Sea Water

**Figure 1:**
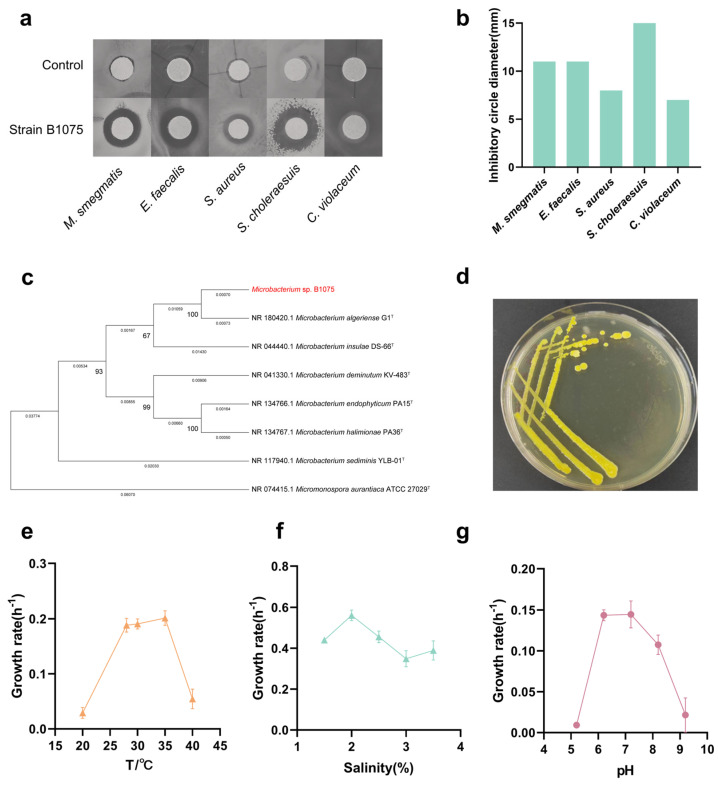
Identifying *Microbacterium* sp. B1075 as the target strain. (**a**) The antibacterial activity of strain B1075 was validated using the K-B paper disk method, with methanol solution added in the blank control group. (**b**) The inhibition zones were measured in two diameters, and the corresponding inhibition values were obtained by taking the average. (**c**) Neighbor-joining tree based on the 16S rRNA gene sequences of *Microbacterium* sp. B1075 and closely related taxa within the *Microbacterium* genus. (**d**) Macroscopic image displaying the colony morphology of *Microbacterium* sp. B1075. (**e**) Growth rates of *strain* B1075 at different temperatures (n = 3). (**f**) Growth rates of strain B1075 at different salinities (n = 3). (**g**) Growth rates of strain B1075 at different pH values (n = 3).

Five bacterial strains were isolated from three water samples collected from the Mariana Trench (Figure S1). Based on morphological identification and 16S rRNA sequence analysis, the isolated strains were classified as *Microbacterium* members: *Microbacterium* sp. B1075, *Microbacterium* sp. B755, *Microbacterium* sp. B423, *Microbacterium* sp. B481 and *Microbacterium* sp. B419 (Table S1 and Figure S1b). To confirm the bioactivity of the five strains, we conducted Kirby–Bauer experiments to assess the antibacterial activity of crude extracts from these isolates against five pathogens. The isolated *Microbacterium* exhibited varying degrees of bioactivity against five pathogenic bacteria, and all five strains of *Microbacterium* showed bioactivity against the *Chromobacterium violaceum* (Figure S1). This suggests that *Microbacterium*, as a secondary metabolite producer of the marine rare actinomycete genus, has high potential to yield biologically active compounds. Among them, strain B1075, displaying strong inhibitory activity and clear zones of inhibition against *Mycobacterium smegmatis*, *Enterococcus faecalis* and *Salmonella choleraesuis* (Figure 1a), was selected as the representative for further study. According to the results shown in Figure 1b, the inhibited diameters of strain B1075 were 11 mm, 11 mm, 8 mm, 15 mm, and 7 mm, respectively (Figure 1b).

Based on the alignment of 16S rRNA gene sequences, the taxonomic information of strain B1075 was determined (Figure 1c). After cultivation on 2216E agar at 28 °C for 3 days, typical morphological characteristics of colonies were observed and recorded for classification. Strain B1075 displayed smooth, round, convex, and yellow-colored colony morphology (Figure 1d) on MA for 2 days at 28 °C. Optimal growth was observed within the temperature range of 20 to 40 °C (Figure 1e), salt concentration range of 1.5% to 3.5% (Figure 1f), and pH range of 5.2 to 9.2 (Figure 1g and Figure S2).

### 2.2. Genome Analysis of Microbacterium sp. B1075

**Figure 2:**
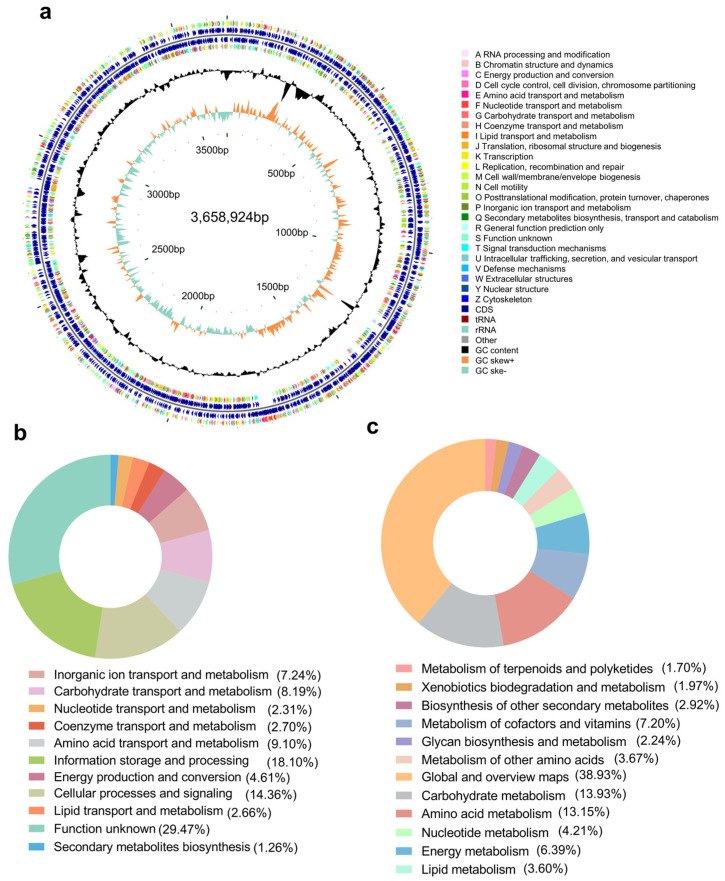
Genome annotation of strain B1075. (**a**) The circular map depicts the CDSs on the positive and negative strands, with different colors indicating different COG functional classifications. The first and fourth circles represent CDSs on the positive and negative strands, respectively, while the second and third circles represent tRNA and rRNA. The fifth circle represents the GC content, the sixth circle represents the GC-Skew value, and the innermost circle indicates the genome size (source: cloud.majorbio.com). (**b**) Pie chart showing the percentage of metabolism-related genes annotated using the COG database for strain B1075. (**c**) Pie chart illustrating the percentage of metabolism-related genes annotated using the KEGG database for strain B1075.

For genome sequencing of *Microbacterium* sp. B1075, a hybrid sequencing approach combining Illumina and PacBio methods was employed, ensuring sequencing depths of not less than 100× for both PacBio and Illumina data to achieve a more comprehensive and accurate assembly. The sequencing data yielded a complete genome size of 3,658,924 bp for strain B1075, with a G + C content of 68.53 mol%. Annotation identified a total of 3523 genes, including 3468 coding sequences, 3 ncRNA, 6 rRNA and 46 tRNA (Figure 2a).

The 3468 protein coding genes constituted 98.4% of the total genes in the genome, but only 80.8% were predicted with functions. Furthermore, there were only 2818 genes (80.0%) assigned to 24 different clusters of orthologous groups (COGs), 1095 genes (31.1%) connected to KEGG pathways, and 842 genes (23.9%) connected to Meta Cyc pathways.

COG (Clusters of Orthologous Groups) annotation revealed 3078 functional proteins, with 38.07% associated with microbial metabolism (Figure 2b). However, approximately 29.47% of the genes were annotated as having unknown functions. Utilizing the KEGG (Kyoto Encyclopedia of Genes and Genomes), 2165 genes were annotated, with metabolic pathways constituting 67.99%, environmental information 8.91%, genetic information processing 8.40%, and cellular processes 8.17% (Figure 2c).

Transport proteins, forming intricate channels, carriers, pumps, and electron carriers, play a crucial role in determining cellular molecular composition and energy status, closely intertwined with microbial metabolism. Functional predictions based on the TCDB (Transporter Classification Database) annotated a total of 629 transport-related genes in strain B1075, including 346 genes for Primary Active Transporters, 159 for Electrochemical Potential-driven Transporters, 5 for Transmembrane Electron Carriers, 37 for Channels/Pores, 20 for Accessory Factors Involved in Transport, 11 for Group Translocators, and 51 for Incompletely Characterized Transport Systems (Figure S3).

Carbohydrate-active enzymes are pivotal in microbial secondary metabolism, orchestrating the breakdown and synthesis of carbohydrates. This regulatory role significantly impacts the types and quantities of secondary metabolites, thereby facilitating the prediction of the genome’s CAZymes (carbohydrate-active enzymes). The results revealed annotations for a total of 96 genes related to carbohydrate-active enzymes, including 35 for Glycoside Hydrolases, 26 for Carbohydrate Esterases, 25 for Glycosyl Transferases, 9 for Auxiliary Activities, and 1 for Carbohydrate-Binding Modules (Figure S3).

### 2.3. GNPS Molecular Network Analysis of Secondary Metabolites

**Figure 3:**
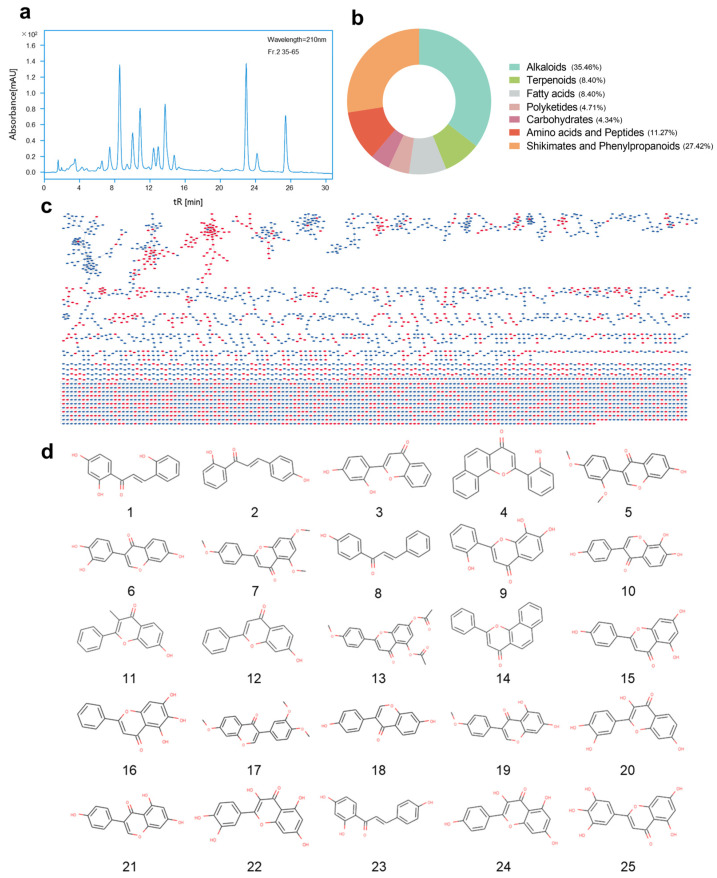
Annotation results of secondary metabolites produced by strain B1075 using GNPS. (**a**) HPLC chromatogram of Fr-2 fraction of secondary metabolites isolated from strain B1075 after separation by ODS column. (**b**) Number of known compounds annotated by GNPS for secondary metabolites Fr-2 produced by strain B1075. (**c**) Molecular network analysis of secondary metabolites Fr-2 produced by strain B1075 using LC-MS/MS technology in positive and negative ion modes. Red nodes represent annotated known compounds, while blue nodes represent unknown compounds. (**d**) Identification of 25 flavonoid compounds (Table S2) annotated by GNPS for Fr-2 fraction.

To conduct a comprehensive and in-depth analysis of the secondary metabolites of *Microbacterium* sp. B1075, we collected mass spectrometry data of the Fr-2 fraction of the fermentation culture in both positive and negative ion modes using UPLC- MS/MS high-resolution mass spectrometry (Figure 3a and Figure S4). Combining with a GNPS network library search online, a total of 3910 nodes and 2262 edges were identified (Figure 3c). The classification of known compounds preliminarily reveals 384 annotations for alkaloids, 122 for amino acids and peptides, 47 for carbohydrates, 91 for fatty acids, 51 for polyketides, 297 for phenylpropanoids, and 91 for terpenoids (Figure 3b). Among phenylpropanoids, there are 25 compounds related to flavonoids, including digitoxin, quercetin, 2′,4′-dihydroxyflavone, 7,8,2′-trihydroxyflavone, and 7-hydroxyflavone, as well as important intermediates in flavonoid synthesis such as chalcone derivatives, like 2,2′,4′-trihydroxychalcone and 2-(4-chlorophenoxy)-1-(2,4-dihydroxyphenyl) ethenone (Figure 3d and Table S2). These classification results provide valuable guidance for further elucidating the potential and medicinal value of flavonoid synthesis in the secondary metabolites of strain B1075.

### 2.4. Fractionation and Purification of Secondary Metabolites Guided by Antibacterial Activity

**Figure 4:**
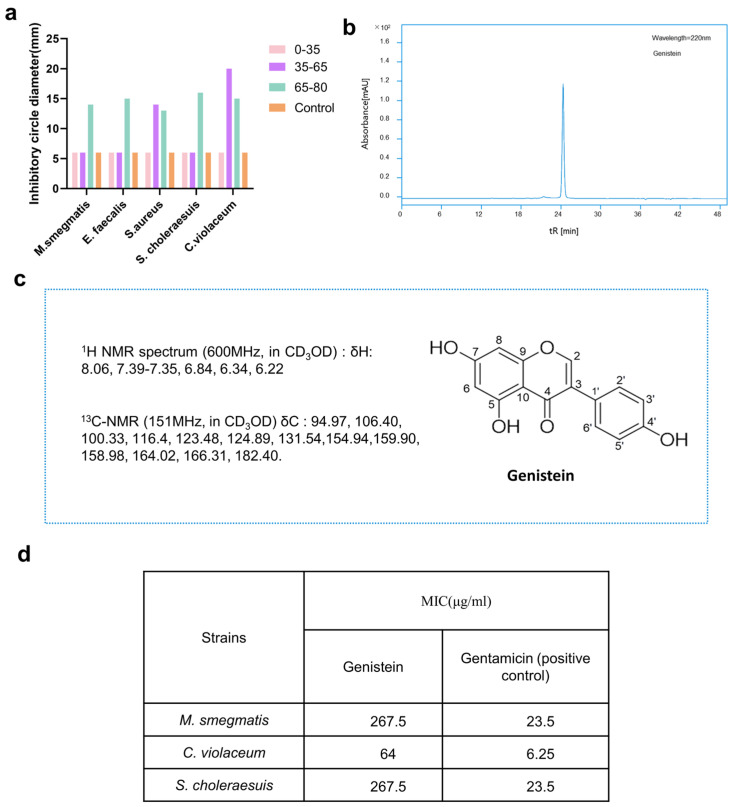
Isolation and identification of secondary metabolites and antibacterial activity of strain B1075. (**a**) Measurement of the diameter of inhibition zones of three fractions of secondary metabolites from strain B1075 separated by an ODS column. Fractions: 0–35 represents Fr-1; 35–65 represents Fr-2; 65–80 represents Fr-3; Control represents ‘control’ group (methanol). (**b**) Chromatogram of compound genistein obtained after the purification of secondary metabolite Fr-2 by HPLC. (**c**) Chemical shifts of carbon and hydrogen spectra along with the molecular structure of the isolated compounds. (**d**) Antibacterial activity values of compound genistein measured using the microdilution method.

The initial antibacterial activity of the crude extract from *Microbacterium* sp. B1075 revealed potential for bioactivity. To further characterize the bioactivity compounds, the ethyl acetate crude extract of strain B1075 was prepared and isolated based on the stepwise antibacterial activity of its sequentially separated secondary metabolites. A total of 1.324 g of crude extract was obtained through fermentation in TSB culture medium. The crude extract was subjected to a bioactivity-guided fractionation strategy, and the segmented activity is illustrated in Figure 4a and Figure S5. Additionally, based on the segmented antibacterial results and the analysis annotations from GNPS (Figure 3a), we proceeded with the purification of compounds from the Fr-2 fraction, which showed better antibacterial activities than the other fractions (Figure 4a). Eventually, a compound with valuable bioactivity, designated as compound **1** (6 mg), was purified. The compound appeared as a pale-yellow powder with an absorption peak at 220 nm (Figure 4b).

The secondary mass spectrometry analysis of compound **1** showed m/z 269.0455 [M − H]^−^ in negative ion mode and *m*/*z* 271.0514 [M + H]^+^ in positive ion mode, with its sodium adduct at *m*/*z* 293.0339 [M + Na]^+^. Based on these data, its relative molecular mass was calculated to be 270.24 g/mol, with a molecular formula of C_15_H_10_O_5_ (Figure S6). Further analysis and comparison of its mass spectrometry data and potential chemical properties were required to distinguish it from the structural isomer. Its 1H NMR spectrum (600 MHz, in CD_3_OD) showed the following signals: δ 8.06 (s, 1H), 7.39–7.35 (m, 2H), 6.84 (d, J = 8.9 Hz, 2H), 6.34 (d, J = 1.8 Hz, 1H), 6.22 (d, J = 2.6 Hz, 1H).^13^C-NMR (151 MHz, in CD_3_OD) δ C: 94.97 (C-8), 106.40 (C-10), 100.33 (C-6), 116.4 (C-3′5′), 123.48 (C-1′), 124.89 (C-3), 131.54 (C-2′6′), 154.94 (C-2), 159.90 (C-9), 158.98 (C-4′), 164.02 (C-5), 166.31 (C-7), 182.40 (C-4). Comparison with carbon and hydrogen spectra databases confirmed the compound as a genistein [19] (Figure 4c and Figure S7).

To further validate the inhibitory activity of genistein against pathogenic bacteria, three strains exhibiting significant inhibition zones in the preliminary assay were chosen as targets for determining the MIC (minimum inhibitory concentration) values of genistein. The results from the microbroth dilution method indicate that the MIC values of genistein against *Mycobacterium smegmatis*, *Chromobacterium violaceum*, and *Salmonella choleraesuis* were 267.5 μg/mL, 64 μg/mL, and 267.5 μg/mL, respectively. In comparison, the MIC values for the positive control gentamicin were 23.5 μg/mL, 6.25 μg/mL, and 23.5 μg/mL (Figure 4d).

### 2.5. Proposed Genistein Biosynthesis Pathway in Microbacterium sp. B1075

**Figure 5:**
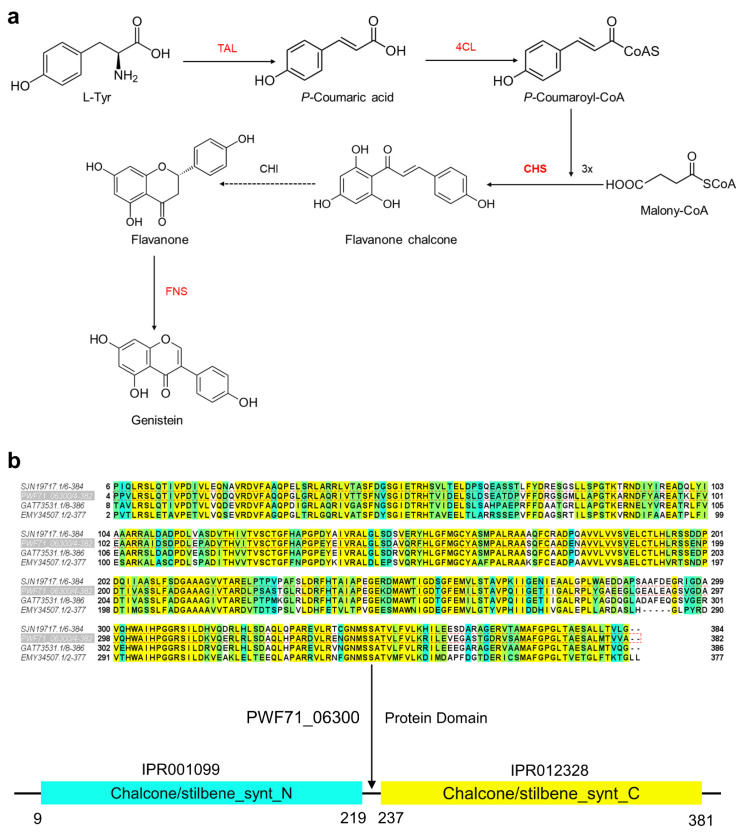
Pathway for the synthesis of flavonoid compounds in strain B1075. (**a**) Enzyme genes in strain B1075 predicted to be associated with the biosynthetic pathway are highlighted in red. Enzyme gene with sequence similarity below 30% is displayed in black, and the catalytic step is indicated by dashed line. TAL, tyrosine ammonia-lyase; 4CL, 4-coumaroyl-CoA ligase; CHS, chalcone synthase; CHI, chalcone isomerase; FNS, flavone synthase. (**b**) Homologous sequences of CHS with similarity exceeding 60% were obtained by amino acid sequence alignment of strain B1075 through NCBI. Among them, the amino acid sequence similarity of GAT73531.1 reached 82.94%, represented by a gradient from yellow to blue, with higher similarity shown in yellow and lower similarity in blue. Protein domain prediction of the CHS fragment (encoded by the gene *CHS*) in strain B1075 was conducted using InterPro. Blue bar represents the N-terminal domain of chalcone and stilbene synthases identified in CHS of B1075. Yellow bar represents the C-terminal domain of chalcone and stilbene synthases identified in CHS of B1075. Graphical representation was generated using Jalview 2.11.3.0.

In the tyrosine pathway of genistein synthesis, tyrosine is initially catalyzed by tyrosine ammonia-lyase (VAL), yielding *p*-coumaric acid, which is further transformed to *p*-coumaroyl-CoA by 4-coumaroyl-CoA ligase (4CL). After that, chalcone synthase (CHS) is responsible for the synthesis of flavanone chalcone by adding 3X malonyl-CoA to *p*-coumaroyl-CoA. Flavanone chalcone is modified by chalcone isomerase (CHI) and converted to flavanone. Finally, genistein is synthesized by flavone synthase (FNS) [20]. Based on the synthesis pathways of flavonoids identified in bacteria [20], we conducted predictive analysis of the synthesis pathway by integrating the whole-genome sequencing results of *Microbacterium* sp. B1075. We conducted a local BLAST search on strain B1075 to identify putative homologous proteins involved in flavonoid biosynthesis. The comparison revealed a 72% protein sequence similarity of PWF71_01175 (WEF21307) to tyrosine ammonia-lyase (TAL, VEH25920) from *Cellulomonas fimi* NCTC7547, a 42% sequence similarity of PWF71_13600 (WEF20308) to 4-coumaroyl-CoA ligase (4CL, AHE41426) from *Scutellaria lateriflora* L, an 82% sequence similarity of PWF71_06300 (WEF22284) to chalcone synthase (CHS, GAT73531) from *Microbacterium* sp. HM58-2, a 29% sequence similarity of PWF71_14570 (WEF20497) to chalcone isomerase (CHI, WP_187272318) from *Zeimonas arvi* CC-CFT501, and a 32% sequence similarity of PWF71_09720 (WEF19579) to flavone synthase (FNS, QCP71067) from *Pohlia nutans* Lindb (Table S3). Based on the BLAST results, the predicted biosynthetic pathway of genistein is depicted in Figure 5a. In addition, structural domain prediction of the corresponding protein sequences in strain B1075 using InterPro [21] revealed complete synthesis capability for all predicted flavonoid synthesis enzymes, except for the chalcone isomerase (CHI) portion, where certain domains were missing (Figure S8). Chalcone synthase (encoded by the gene *CHS*, PWF71_06300, 1161 bp), predicted to contain both the N- and C-terminal domains of chalcone/stilbene synthase (Figure 5b), plays a pivotal role by condensing one molecule of 4-coumaroyl-CoA with three molecules of malonyl-CoA to produce the chalcone of genistein (Figure 5a). The generated chalcone serves as the carbon skeleton for the in vivo synthesis of flavonoids, providing a material basis for the synthesis of isoflavones, flavones, anthocyanin glycosides, flavanols, and other flavonoid substances [22].

### 2.6. Regulation of Key Genes of Flavonoids under High Pressure

**Figure 6:**
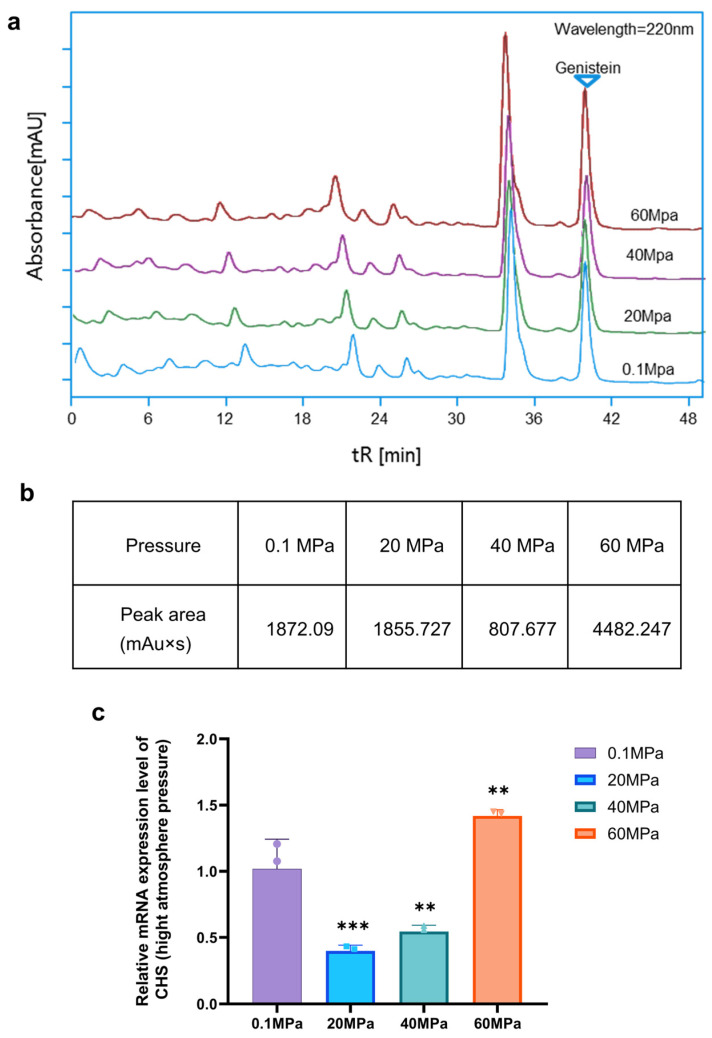
Impact of high hydrostatic pressure on the production of flavonoid compounds by strain B1075. (**a**) HPLC chromatograms of secondary metabolites extracted from strain B1075 under different hydrostatic pressure conditions. (**b**) Peak areas of genistein produced by strain B1075 at different pressures. The peak area at 60 MPa is approximately twice that at 0.1 MPa. Data represent the mean values of three independent experiments with similar results. (**c**) Relative mRNA expression level of the gene *CHS* under different pressure conditions. Error bars indicate the standard deviations from three independent experiments (*** *p* < 0.001, ** *p* < 0.01).

To explore the impact of the extreme abyssal environment on the secondary metabolites of *Microbacterium* sp. B1075, we subjected seed liquid to cultivation under high-pressure conditions of 0.1 MPa, 20 MPa, 40 MPa, and 60 MPa for 5 days. After measuring the OD values to calculate an equal number of cells, we inoculated them into TSB liquid culture to ferment and extract secondary metabolites. We then analyzed the effects of high-pressure conditions on the production of genistein by strain B1075 using high-performance liquid chromatography (HPLC) (Figure 6a). The peak areas of the compound genistein showed alterations when stimulated by different high-pressure conditions, with the peak areas showing an increase under 60 MPa relative to 0.1 MPa (Figure 6b).

To investigate the impact of high pressure on the production of genistein in strain B1075, qRT-PCR primers qCHSF1/qCHSR1 were designed using Primer6 (Table S4). Total RNA was extracted from cells cultivated under pressures of 0.1 MPa, 20 MPa, 40 MPa, and 60 MPa, and qRT-PCR was employed to examine the effect of different pressures on the expression levels of the gene *CHS*. All measurements were taken using three biological replicates and technical replicates. Pairwise comparisons were performed using *t*-tests. The results indicate that high-pressure stimulation primarily affects the expression of the gene *CHS* at 60 MPa. Relative to the strain under 0.1 MPa, the expression level of the gene *CHS* increased by 1.5-fold under the high-pressure environment of 60 MPa, demonstrating a significant upregulation effect of high-pressure stimulation on the expression of the gene *CHS* (**** *p* < 0.0001) (Figure 6c).

## 3. Discussion

Research on secondary metabolites produced by actinomycetes in marine environments has demonstrated vast potential applications [3]. The extreme conditions of the deep-sea environment (>5000 m) pose significant challenges to microbial survival, necessitating adaptation through primary and secondary metabolic pathways. It is noteworthy that secondary metabolites isolated from deep-sea environments exhibit remarkably high biological activity [23]. However, there are still several issues worthy of further investigation. Diversity analysis of rare actinomycetes reveals extraordinarily complex patterns of secondary metabolite production. Accelerating the research process involves molecular studies and compound identification screening to advance the application of these secondary metabolites in drug discovery [24]. *Microbacterium*, as a rare genus of actinomycetes, has received relatively limited attention in research. However, existing studies indicate its enormous potential. For instance, some studies have focused on its adaptation mechanisms in the deep-sea environment, exploring its cold and pressure tolerance characteristics [25,26], as well as the production of its linear peptide-like active substances [27,28], and its application in the degradation of heavy metals and pollutant decomposition [10,11].

In this study, we targeted the screening of *Microbacterium* species based on their antibacterial activity and obtained the desired strain for further investigation. We conducted extensive isolation of its secondary metabolites, focusing particularly on the elucidation of the biosynthetic pathway of the isolated compound, genistein, within strain B1075. Genistein, as an active flavonoid compound, holds significant pharmaceutical value, predominantly explored in leguminous plants [29,30], undergoing glycosylation modification within the plant’s biosynthetic pathway. Although the biosynthetic pathways for isoflavones have been identified in actinobacteria, particularly in the genus *Streptomyces* [31], previous research has primarily focused on biosynthesis or glycosylation modifications. Despite the isolation of glycosylated isoflavones, such as glycosylated genistein, from terrestrial-derived *Microbacterium* species [32], there are structural differences compared to the unglycosylated genistein isolated in this study, and further genomic analysis is lacking in previous studies. Additionally, research on the regulation of isoflavone biosynthesis pathways in *Streptomyces* by environmental factors is also quite limited.

It is noteworthy that the secondary metabolites of strain B1075 demonstrated the capability to synthesize flavonoids, even without the addition of specific precursor substances. Through the predictive analysis from the GNPS platform, we identified not only the potential to produce genistein but also possibly superior synthetic abilities compared to other actinomycetes genera producing flavonoids. Additionally, by integrating conventional microbial pathways for flavonoid synthesis with the complete genomic information of strain B1075, we predicted the relevant enzyme genes involved in synthesizing flavonoids within the strain. The results indicated the full capacity of strain B1075 to synthesize flavonoid compounds. The bidirectional validation between bioinformatics predictions and actual secondary metabolite identification in strain B1075 further corroborated its value in producing secondary metabolites as a deep-sea Actinomycete. This not only enriches the diversity of flavonoid synthesis in actinomycetes but also offers new insights for utilizing microbial production of flavonoids.

High pressure is a prevalent feature in the deep-sea environment, and previous studies have indicated that DNA and protein synthesis are the most pressure-sensitive processes when examining the relationship between DNA, RNA, protein synthesis, and pressure [33]. This is closely related to the secondary metabolism of microorganisms in the deep sea. Additionally, we investigated the connection between strain B1075, a deep-sea Actinomycete, and its surrounding deep-sea environment. Consequently, we targeted the key biosynthetic gene *CHS* responsible for producing genistein and investigated its response to high-pressure conditions in the deep-sea environment. We observed the effects of high-pressure conditions on the production of flavonoid compounds by strain B1075. At pressures of 20 MPa and 40 MPa, high-pressure conditions suppressed the expression of the gene *CHS* and decreased product yield. However, under in situ conditions at 60 MPa, the expression of the gene *CHS* was significantly upregulated, and product yield also increased comparing to normal atmospheric pressure. Overall, the expression level of the gene *CHS* shows a trend of initially decreasing and then increasing compared to normal-pressure conditions, which warrants further investigation. Additionally, we observed corresponding supplementary results at the metabolite level, indicating a positive effect of in situ high-pressure cultivation on the secretion of genistein by strain B1075. High-pressure conditions may induce significant metabolic changes within cells by altering the concentration of dissolved gases and the cellular oxidation state [34,35]. In response to such extreme conditions, antioxidant defense mechanisms have been shown to be crucial for cell defense [34,36]. Given that previous studies have demonstrated the excellent antioxidant properties of genistein [37], the increased production of genistein in strain B1075 under in situ pressure conditions may provide a protective mechanism for the deep-sea environment. The other flavonoids (Figure 3d) detected in strain B1075 may also contribute to the high-pressure adaption, which warrants further investigation in future. This is the first time that the production of flavonoid compounds by actinomycetes has been speculatively linked to high-pressure environmental stress. However, this remains an issue worthy of further exploration. Our research provides clear evidence for this question, emphasizing the close relationship between deep-sea microorganisms and their extreme environment. This contributes to a more comprehensive understanding of the survival strategies of microorganisms in the deep-sea environment and the regulation mechanisms of their secondary metabolism.

In summary, our study strengthens the exploration and analysis of secondary metabolites from rare Actinobacteria of the genus *Microbacterium* in the ocean. Through our preliminary exploration of strain B1075, we have tentatively identified its potential as a candidate for natural bioactive compounds. We successfully isolated the genistein from deep-sea *Microbacterium* and proposed a pathway for the biosynthesis of flavonoids in strain B1075. This work suggests the possibility of marine *Microbacterium* as a source of natural active metabolites, which warrants further investigation. Furthermore, our research has begun to explore the relationship between extreme deep-sea environments and microbial metabolism. Our initial findings hint at the influence of high pressure on the production of microbial secondary metabolites, an area that deserves more attention. Our study offers a modest contribution to the understanding of secondary metabolic mechanisms in deep-sea microorganisms and can serve as a foundation for future research in drug discovery and the development of natural products, acknowledging that there is much more to explore in this vast and complex field.

## 4. Materials and Methods

### 4.1. Sample Collection and Strain Isolation

Sea water samples were collected from a 5900 m depth at station MBR01 of the Mariana Trench (11°40.38′ N, 142°6.16′ E) during the TS15 cruise of the research vessel ‘Tan Suo Yi Hao’ in November 2019, by using Niskin bottles fitted on a Sea-Bird Carousel equipped with a CTD sensor (Sea-Bird Scientific, Bellevue, WA, USA). After being brought on board, the sea water samples were immediately subsampled and stored in 50 mL sterile centrifuge tubes at 4 °C. Half-strength marine agar 2216 (MA; BD Difco, Franklin Lakes, NJ, USA) was used to isolate culturable bacteria by using the standard dilution plating technique at 28 °C. Stock cultures were stored at −80 °C with 20% (*v*/*v*) glycerol (Table S5).

### 4.2. 16S rDNA Cloning and Sequencing for Bacterial Identification

Genomic DNA for 16S rRNA gene cloning was extracted from bacteria cells using the Rapid Bacterial Genomic DNA Isolation Kit (Sangon, Shanghai, China) according to the manufacturer’s instructions. The 16S rRNA gene was PCR-amplified and -sequenced by using conserved primers 27F (5′-AGAGTTTGATCATGGCTCAG-3′) and U1492R (5′-GGTTACCTTGTTACGACTT-3′) [38]. The PCR reaction program was as follows: pre-denaturation at 95 °C for 120 s, followed by denaturation at 95 °C for 15 s, annealing at 55 °C for 30 s, and extension at 72 °C for 90 s. Steps 2 to 4 were repeated for 30 cycles, and a final extension was performed at 72 °C for 10 min. Pairwise 16S rRNA gene sequence similarity was determined using the EzBioCloud server (https://www.ezbiocloud.net/identify, accessed on 6 May 2024) [39]. Phylogenetic analysis of 16S rRNA gene was performed using the software MEGA X 10.2 [40]. Distances were calculated using the Kimura two-parameters model and clustering was performed with the neighbor-joining algorithm [41]. The robustness of the inferred topology was calculated by bootstrap analysis based on 1000 replications.

### 4.3. Genomic DNA Preparation and Whole-Genome Sequencing

Genomic DNA for genome sequencing was extracted from liquid cultures after being cultivated in SOB medium for 36 h using the ChargeSwitch^®^ gDNA Mini Bacteria Kit (Thermo Fisher Scientific, Wilmington, DE, USA) according to the manufacturer’s instructions. The complete genome was sequenced using a combination of Illumina Hiseq and Pacific Biosciences (PacBio, Menlo Park, CA, USA) RS sequencing platforms (Shanghai Majorbio Bio-pharm Technology Co., Ltd., Shanghai, China). For Illumina sequencing, a 400 bp paired-end library was generated and sequenced using Illumina Hiseq X Ten. For PacBio sequencing, 8–10 k insert whole-genome shotgun libraries were generated and sequenced on a PacBio RS instrument using standard methods. The genome was assembled using SOAPdenovo (version 2.04) and Unicycler (version 0.5.0) software, and sequence correction was performed using Pilonjin (version 1.14) software. The complete genome sequence of *Microbacterium* sp. B1075 was deposited in the GenBank database under the accession number CP118606.

### 4.4. RNA Extraction and Quantitative Real-Time PCR

Total RNA was isolated using RNAiso Plus (TaKaRa, Dalian, China) according to the manufacturer’s instructions. Then, cDNA was synthesized using the PrimeScript RT reagent kit with gDNA Eraser (Perfect Real Time) (TaKaRa, Dalian, China). The concentration of RNA was then quantified using a Nano-Drop 2000c spectrophotometer (Thermo Fisher Scientific, Wilmington, DE, USA). The qRT-PCR was performed using SYBR Premix Ex Taq II (Tli RNaseH Plus) (TaKaRa, Dalian, China) on the ABI 7500 Real Time PCR System (Thermo Fisher Scientific, Wilmington, DE, USA). The reactions were performed using the following conditions: initial denaturation at 95 °C for 30 s, followed by 40 cycles of 95 °C for 5 s, and 60 °C for 34 s. 16s rRNA gene was chosen as an internal reference gene for normalization and the fold changes were calculated using the formula 2^−ΔΔCT^. The qRT-PCR was performed for triplicate independent experiments. Significance analysis was performed using ordinary one-way ANOVA with GraphPad Prism 8.0.3.

### 4.5. Extraction of Secondary Metabolites

Bacterial strain *Microbacterium* sp. B1075 was inoculated into 100 mL of sterile TSB medium and incubated at 28 °C for 18 h on a rotary shaker (180 rpm) to produce a seed broth. The seed broth (1 mL) was transferred to a 500 mL conical flask containing 200 mL of TSB liquid culture medium. The conical flasks were incubated at 28 °C for 5 days. A total of 25 flasks were prepared in this study. The liquid culture of *Microbacterium* sp. B1075 was extracted three times with ethyl acetate (1:1, *v*/*v*). The upper layer of the ethyl acetate extract was taken from the conical flasks and dried using a rotary evaporator to obtain crude extracts for subsequent analysis.

For high-pressure cultivation, 20 mL of cell cultures at exponential growth phase (OD_600_ = 0.5) was transferred to a high-pressure bag (ProAmpac, Cincinnati, OH, USA) and cultivated in hydrostatic pressure vessels at 0.1, 20, 40, and 60 MPa at 28 °C for 3 days in triplicates. After high-pressure cultivation, the seed broth was inoculated to a 500 mL conical flask containing 200 mL of TSB liquid culture medium and incubated at 28 °C and 180 rpm for 5 days. Different volumes of liquid cultures but equal cell numbers (OD_600_ based) were used for the extraction of secondary metabolites and HPLC analysis.

### 4.6. UPLC-MS/MS Analysis of Secondary Metabolites

The crude extract (1.358 g) was fractionated by a Octadecyl-functionalized silica gel column (ODS, Cosmosil 75 C_18_-Prep, 30 × 500 mm, 75 µm) with MeOH/H_2_O (3.5:6.5, 6.5:3.5, 8:2, *v*/*v*) to furnish three fractions (Fr-1-Fr-3) based on the results of HPLC analysis. Fr-2 (0.2078 g) was further purified by Auno lc-2000 using a column with methanol and water elution solvent (Auno pre-column C_18_, 30 × 250 mm, 10 μm; flow rate: 15 mL/min; UV detection: 220 nm) with MeOH/H_2_O (90:10, *v*/*v*) to afford four subfractions (Fr-2.1-2.4).

The characterization of Fr-2 crude extract from *Microbacterium* sp. B1075 was performed by ultra-high-pressure liquid chromatography/tandem mass spectrometry (UPLC-MS/MS). The crude extract was dissolved in 1 mL of methanol and filtered through a 0.22 μm filter. UPLC-MS/MS spectra were acquired on a Vanquish UPLC high-resolution mass spectrometer (Thermo Fisher Scientific, Wilmington, DE, USA) equipped with an electrospray ionization (ESI) source that can be operated in positive and negative ionization modes. A Hypersil Gold C_18_ column (2.1 mm × 150 mm, 19 µm) was used at a flow rate of 0.4 mL/min, with a column temperature of 40 °C and an injection temperature of 10 °C.

LC-ESI-MS/MS mass spectrometry, GNPS molecular network and MolEnhancer [42] techniques were applied to characterize the chemical composition of the crude extract of the Fr-2 fraction of *Microbacterium* sp. B1075.

A molecular network was created using the online workflow on the GNPS website (https://ccms-ucsd.github.io/GNPSDocumentation/, accessed on 21 November 2023). The precursor ion mass tolerance was set to 2.0 Da and a MS/MS fragment ion tolerance of 0.5 Da. A network was then created where edges were filtered to have a cosine score above 0.65 and more than 6 matched peaks. Further, edges between two nodes were kept in the network if and only if each of the nodes appeared in each other’s respective top 10 most similar nodes. Finally, the maximum size of a molecular family was set to 100, and the lowest-scoring edges were removed from molecular families until the molecular family size was below this threshold. The spectra in the network were then searched against GNPS spectral libraries. All matches kept between network spectra and library spectra were required to have a score above 0.7 and at least 6 matched peaks.

### 4.7. Isolation and Identification of Compounds

Compound **1** (6 mg, tR 24 min, MeOH/H_2_O, 96/4) was purified from Fr-2.4 by Agilent 1260 HPLC using a column (Agilent Zorbax SB-C_18_, 250 × 9.4 mm, 5 μm; flow rate: 1 mL/min; UV detection: 220 nm). HPLC and analytic grace solvents used for this study were purchased from Sinopharm Chemical Reagent Co., Ltd. (Shanghai, China).

Optical rotation was recorded on a RUDOLPH Autopol I Automatic polarimeter (Rudolph Research Analytical). NMR spectra were acquired on a Bruker 500 spectrometer or a JEOL 600 spectrometer using the standard programs and acquisition parameters, and the chemical shifts were expressed in δ (ppm) relative to CD_3_OD (δC 49.00 and δH 3.31).

### 4.8. Antimicrobial Activity Assay

The indicator bacteria used in the bacteriostatic circle experiment were provided by Shanghai Rainbowfish Company, including *Chromobacterium violaceum* ATCC 12472, *Salmonella choleraesuis* subsp. Typhimurium DT104, *Mycobacterium smegmatis* MC2155, *Enterococcus faecalis* FA2-2, and *Staphylococcus aureus* ATCC25923. Cultures of five indicator bacteria (with an OD of approximately 0.5) were inoculated in LB medium in an amount of 100 μL. Sterilized circular filter paper sheets were attached to the LB medium. We dissolved 100 mg of the crude extract in 1 mL of methanol. Then, 6 μL of the crude extracted metabolites and negative control (methanol) was dropped onto the circular filter paper sheet after being incubated at 37 °C for 12 h. Antibacterial bioactivity was evaluated by measuring the area of the zone inhibition and calculating the inhibition rate.

The micro-broth dilution method as described previously was used to further determine the minimum inhibitory concentrations (MICs) [43]. A total 2 μL of a 2-fold serial dilution of compound (in DMSO) was added to each row on a 96-well microplate containing 100 μL of pathogens suspension in each well. Gentamicin was used as a positive control and DMSO was used as a negative control. The 96-well plate was incubated at 37 °C aerobically for 24 h.

Pie charts and bar graphs of inhibition zones, growth rates, and COG and KEGG categories were made using GraphPad Prism 8.0.3. 

## Data Availability

The data presented in the study are deposited in the National Center for Biotechnology Information (NCBI, https://www.ncbi.nlm.nih.gov/, accessed on 8 March 2023) repository. The entire whole-genome gene sequence dataset in this paper has been uploaded to the NCBI Sequence Read Archive (accession no. CP118606).

## Supplementary Materials

The following supporting information can be downloaded at: https://www.mdpi.com/article/10.3390/md22060276/s1, Figure S1: Sample sites information and the antibacterial effect of five strains; Figure S2: The optimal growth conditions of *Microbacterium* sp. B1075; Figure S3: Types of CAZymes and transport proteins in *Microbacterium* sp. B1075; Figure S4: Secondary mass spectrometry analysis of secondary metabolites from *Microbacterium* sp. B1075, fraction Fr-2; Figure S5: Purification and Antibacterial Activity of Secondary Metabolites from *Microbacterium* sp. B1075; Figure S6: TOF-MS spectrum of genistein; Figure S7: Nuclear magnetic resonance spectroscopy of genistein; Figure S8: Predicting the structural domains of enzymes involved in flavonoid biosynthesis in *Microbacterium* sp. B1075 using InterPro; Table S1: Identification of 5 purified bacteria isolated from different depths in Mariana Trench based on 16S rRNA gene sequence analyses; Table S2: The secondary metabolites of *Microbacterium* sp. B1075, fraction Fr-2, were identified to contain 25 flavonoid compounds through secondary mass spectrometry data analysis in the GNPS database; Table S3: Alignment results of the enzyme genes related to genistein biosynthesis in strain B1075; Table S4: Primers required for the experiment; Table S5: Formulation and pH Data of Media.

## References

[1] Ribeiro I., Antunes J.T., Alexandrino D.A.M., Tomasino M.P., Almeida E., Hilário A., Urbatzka R., Leão P.N., Mucha A.P., Carvalho M.F. (2023). Actinobacteria from Arctic and Atlantic deep-sea sediments—Biodiversity and bioactive potential. Front. Microbiol..

[2] Liu R., Wang Z., Wang L., Li Z., Fang J., Wei X., Wei W., Cao J., Wei Y., Xie Z. (2020). Bulk and Active Sediment Prokaryotic Communities in the Mariana and Mussau Trenches. Front. Microbiol..

[3] Wang C., Lu Y., Cao S. (2020). Antimicrobial compounds from marine actinomycetes. Arch. Pharm. Res..

[4] Subramani R., Aalbersberg W. (2013). Culturable rare Actinomycetes: Diversity, isolation and marine natural product discovery. Appl. Microbiol. Biotechnol..

[5] Kamjam M., Sivalingam P., Deng Z., Hong K. (2017). Deep Sea Actinomycetes and Their Secondary Metabolites. Front. Microbiol..

[6] Al-Fadhli A.A., Threadgill M.D., Mohammed F., Sibley P., Al-Ariqi W., Parveen I. (2022). Macrolides from rare actinomycetes: Structures and bioactivities. Int. J. Antimicrob. Agents.

[7] Ding T., Yang L.-J., Zhang W.-D., Shen Y.-H. (2019). The secondary metabolites of rare actinomycetes: Chemistry and bioactivity. RSC Adv..

[8] Subramani R., Sipkema D. (2019). Marine Rare Actinomycetes: A Promising Source of Structurally Diverse and Unique Novel Natural Products. Mar. Drugs.

[9] Liu Y., Chen S., Xie Z., Zhang L., Wang J., Fang J. (2023). Influence of Extremely High Pressure and Oxygen on Hydrocarbon-Enriched Microbial Communities in Sediments from the Challenger Deep, Mariana Trench. Microorganisms.

[10] Corretto E., Antonielli L., Sessitsch A., Höfer C., Puschenreiter M., Widhalm S., Swarnalakshmi K., Brader G. (2020). Comparative Genomics of *Microbacterium* Species to Reveal Diversity, Potential for Secondary Metabolites and Heavy Metal Resistance. Front. Microbiol..

[11] Zhang M., Fan D., Pan L., Su C., Li Z., Liu C., He Q. (2023). Characterization and removal mechanism of a novel enrofloxacin-degrading microorganism, *Microbacterium proteolyticum* GJEE142 capable of simultaneous removal of enrofloxacin, nitrogen and phosphorus. J. Hazard. Mater..

[12] Sohn S.I., Pandian S., Oh Y.J., Kang H.J., Cho W.S., Cho Y.S. (2021). Metabolic Engineering of Isoflavones: An Updated Overview. Front. Plant Sci..

[13] Goh Y.X., Jalil J., Lam K.W., Husain K., Premakumar C.M. (2022). Genistein: A Review on its Anti-Inflammatory Properties. Front. Pharmacol..

[14] Das S., Rosazza J.P.N. (2006). Microbial and Enzymatic Transformations of Flavonoids. J. Nat. Prod..

[15] Wang J.-F., Liu S.-S., Song Z.-Q., Xu T.-C., Liu C.-S., Hou Y.-G., Huang R., Wu S.-H. (2020). Naturally Occurring Flavonoids and Isoflavonoids and Their Microbial Transformation: A Review. Molecules.

[16] Meng Y., Liu X., Zhang L., Zhao G.-R. (2022). Modular Engineering of Saccharomyces cerevisiae for De Novo Biosynthesis of Genistein. Microorganisms.

[17] Falcone Ferreyra M.L., Rius S., Casati P. (2012). Flavonoids: Biosynthesis, biological functions, and biotechnological applications. Front. Plant Sci..

[18] Reimold U., Kröger M., Kreuzaler F., Hahlbrock K. (1983). Coding and 3′ non-coding nucleotide sequence of chalcone synthase mRNA and assignment of amino acid sequence of the enzyme. EMBO J..

[19] Mazurek A.P., Kozerski L., Sadlej J., Kawę R., Bednarek E., Sitkowski J., Dobrowolski J.C., Maurin J.K., Biniecki K., Witowska J. (1998). Genistein complexes with amines: Structure and properties. J. Chem. Soc. Perkin Trans. 2.

[20] Álvarez-Álvarez R., Botas A., Albillos S.M., Rumbero A., Martín J.F., Liras P. (2015). Molecular genetics of naringenin biosynthesis, a typical plant secondary metabolite produced by *Streptomyces clavuligerus*. Microb. Cell Fact..

[21] Paysan-Lafosse T., Blum M., Chuguransky S., Grego T., Pinto B.L., Salazar G.A., Bileschi M.L., Bork P., Bridge A., Colwell L. (2023). InterPro in 2022. Nucleic Acids Res..

[22] Sheng H., Sun X., Yan Y., Yuan Q., Wang J., Shen X. (2020). Metabolic Engineering of Microorganisms for the Production of Flavonoids. Front. Bioeng. Biotechnol..

[23] Skropeta D., Wei L. (2014). Recent advances in deep-sea natural products. Nat. Prod. Rep..

[24] Subramani R., Aalbersberg W. (2012). Marine actinomycetes: An ongoing source of novel bioactive metabolites. Microbiol. Res..

[25] Qiu X., Cao X., Jian H., Wu H., Xu G., Tang X. (2022). Transcriptomic Analysis Reveals that Changes in Gene Expression Contribute to *Microbacterium sediminis* YLB-01 Adaptation at Low Temperature Under High Hydrostatic Pressure. Curr. Microbiol..

[26] Xia J.-M., Hu X.-M., Huang C.-H., Yu L.-B., Xu R.-F., Tang X.-X., Lin D.-H. (2020). Metabolic profiling of cold adaptation of a deep-sea psychrotolerant *Microbacterium sediminis* to prolonged low temperature under high hydrostatic pressure. Appl. Microbiol. Biotechnol..

[27] Liu D., Lin H., Proksch P., Tang X., Shao Z., Lin W. (2015). Microbacterins A and B, New Peptaibols from the Deep Sea Actinomycete *Microbacterium sediminis* sp. nov. YLB-01(T). Org. Lett..

[28] Vitale G.A., Scarpato S., Mangoni A., D’Auria M.V., Della Sala G., De Pascale D. (2023). Enhanced Molecular Networking Shows *Microbacterium* sp. V1 as a Factory of Antioxidant Proline-Rich Peptides. Mar. Drugs.

[29] Jung W., Yu O., Lau S.-M.C., O’Keefe D.P., Odell J., Fader G., McGonigle B. (2000). Identification and expression of isoflavone synthase, the key enzyme for biosynthesis of isoflavones in legumes. Nat. Biotechnol..

[30] Liu R., Hu Y., Li J., Lin Z. (2007). Production of soybean isoflavone genistein in non-legume plants via genetically modified secondary metabolism pathway. Metab. Eng..

[31] Jrumg Z.-D., Fenieal P.J.W. (1997). Actinellavosiae, A Novel Flavonoid-Like Glycoside Produced by a Marine Becterium of the Genus Streptomyces. Tetrahedron Lett..

[32] Savi D.C., Shaaban K.A., Gos F.M.W., Thorson J.S., Glienke C., Rohr J. (2019). Secondary metabolites produced by *Microbacterium* sp. LGMB471 with antifungal activity against the phytopathogen Phyllosticta citricarpa. Folia Microbiol..

[33] Bartlett D.H., Lauro F.M., Eloe E.A. (2007). Microbial Adaptation to High Pressure. Physiology and Biochemistry of Extremophiles.

[34] Xie Z., Jian H., Jin Z., Xiao X. (2018). Enhancing the Adaptability of the Deep-Sea Bacterium Shewanella piezotolerans WP3 to High Pressure and Low Temperature by Experimental Evolution under H_2_O_2_ Stress. Appl. Environ. Microbiol..

[35] Yang S., Lv Y., Liu X., Wang Y., Fan Q., Yang Z., Boon N., Wang F., Xiao X., Zhang Y. (2020). Genomic and enzymatic evidence of acetogenesis by anaerobic methanotrophic archaea. Nat. Commun..

[36] Xiao X., Zhang Y., Wang F. (2021). Hydrostatic pressure is the universal key driver of microbial evolution in the deep ocean and beyond. Environ. Microbiol. Rep..

[37] Sharifi-Rad J., Quispe C., Imran M., Rauf A., Nadeem M., Gondal T.A., Ahmad B., Atif M., Mubarak M.S., Sytar O. (2021). Genistein: An Integrative Overview of Its Mode of Action, Pharmacological Properties, and Health Benefits. Oxidative Med. Cell. Longev..

[38] Turner S., Pryer K.M., Miao V.P., Palmer J.D. (1999). Investigating deep phylogenetic relationships among cyanobacteria and plastids by small subunit rRNA sequence analysis. J. Eukaryot. Microbiol..

[39] Yoon S.-H., Ha S.-M., Kwon S., Lim J., Kim Y., Seo H., Chun J. (2017). Introducing EzBioCloud: A taxonomically united database of 16S rRNA gene sequences and whole-genome assemblies. Int. J. Syst. Evol. Microbiol..

[40] Kumar S., Stecher G., Li M., Knyaz C., Tamura K. (2018). MEGA X: Molecular Evolutionary Genetics Analysis across Computing Platforms. Mol. Biol. Evol..

[41] Saitou N., Nei M. (1987). The neighbor-joining method: A new method for reconstructing phylogenetic trees. Mol. Biol. Evol..

[42] Wang M., Carver J.J., Phelan V.V., Sanchez L.M., Garg N., Peng Y., Nguyen D.D., Watrous J., Kapono C.A., Luzzatto-Knaan T. (2016). Sharing and community curation of mass spectrometry data with Global Natural Products Social Molecular Networking. Nat. Biotechnol..

[43] Xin W., Ye X., Yu S., Lian X.-Y., Zhang Z. (2012). New Capoamycin-Type Antibiotics and Polyene Acids from Marine Streptomyces fradiae PTZ0025. Mar. Drugs.

