# Supplementary material for: Characterization and Biosynthetic Regulation of Isoflavone Genistein in Deep-Sea Actinomycetes Microbacterium sp. B1075
Source: Mar Drugs. 2024 Jun 13;22(6):276. doi: 10.3390/md22060276 (PMC11205022; doi:10.3390/md22060276)
Supplement: Supplementary file 1 [file marinedrugs-22-00276-s001.zip › marinedrugs-3024680-supplementary.pdf]

Supplementary Materials

a

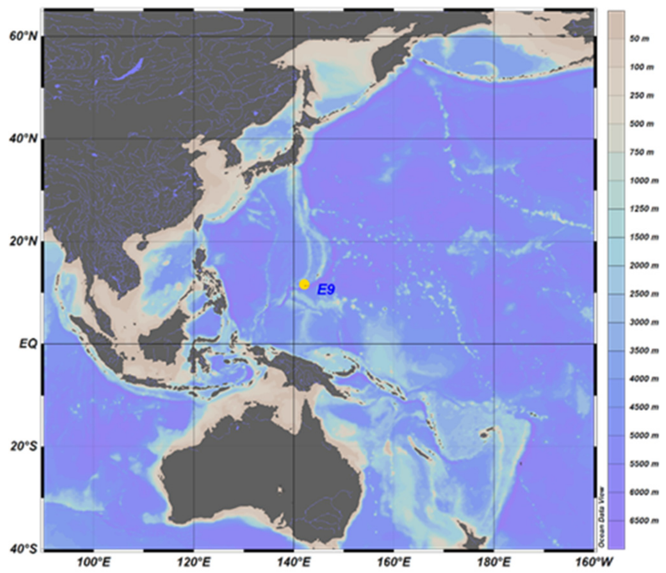

b

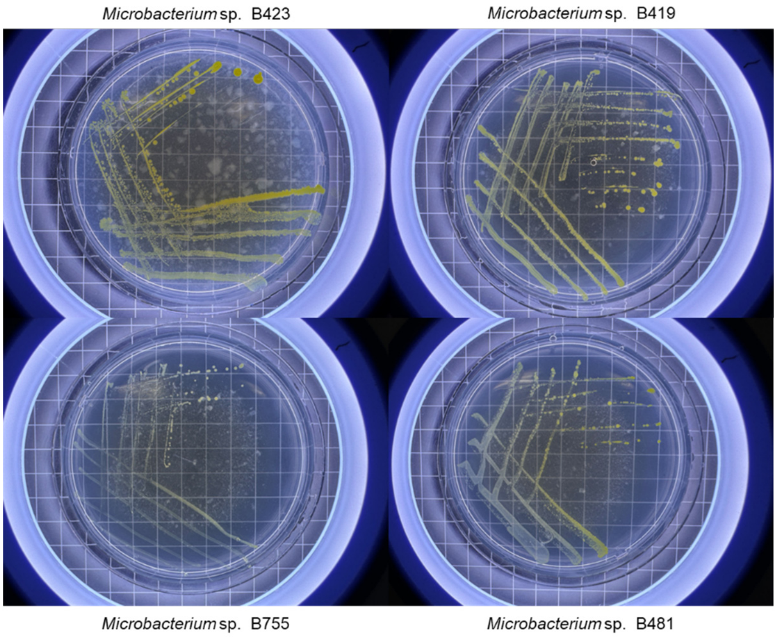

c

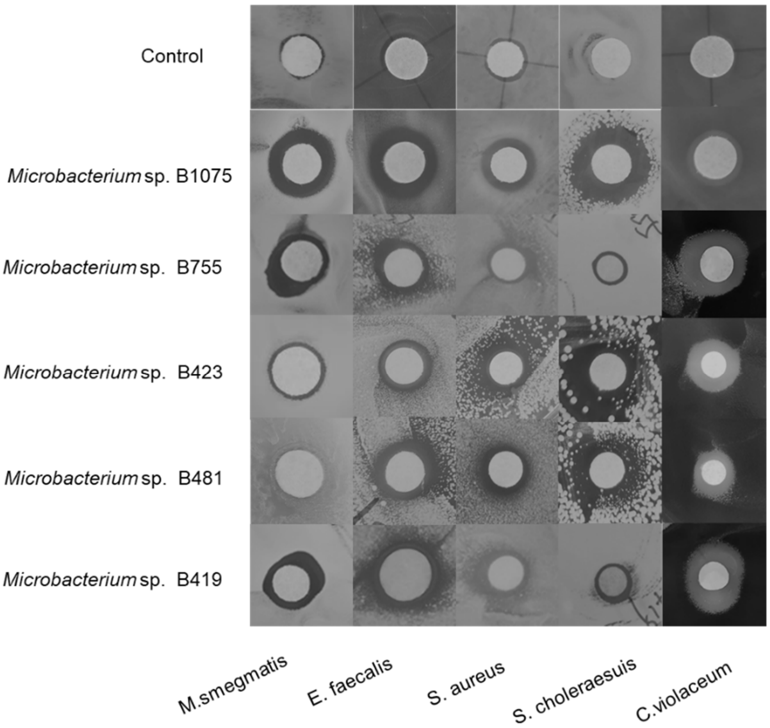

Figure S1. Sample sites information and the antibacterial effect of five strains. (a) The sampling sites of seawater used in this study (11°40.38′N, 142°6.16′E), mapped with ODV 5.6.7. (b) Colony morphology of the four *Microbacterium* strains. (c) The antibacterial activity results of five strains. *Microbacterium* sp. B1075 exhibited the highest inhibitory activity.

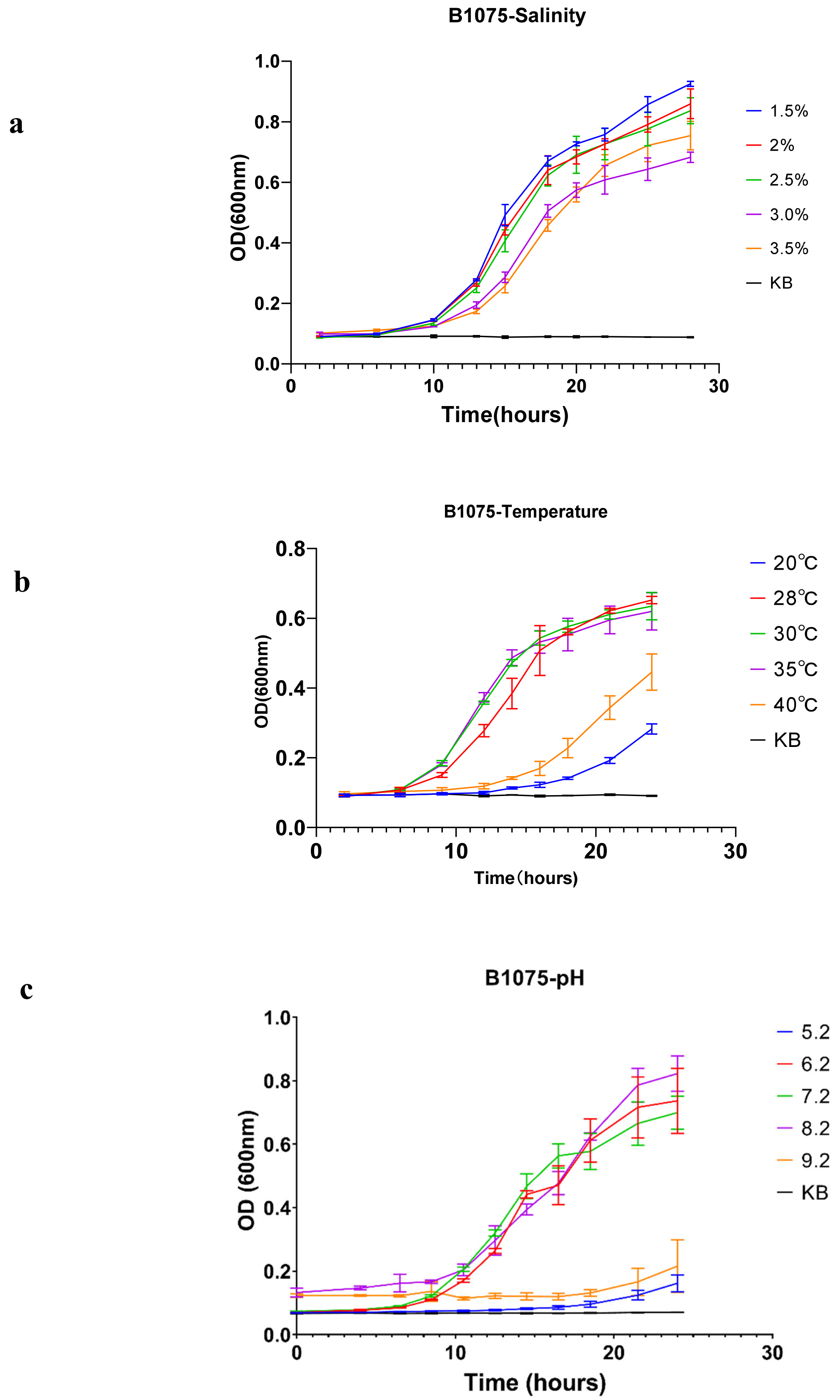

Figure S2. The optimal growth conditions of *Microbacterium* sp. B1075. (a) Growth curves under different salinity. (b) Growth curves under different temperature. (c) Growth curves under different pH values. The term 'KB' refers to the blank medium control without the addition of bacterial suspension.

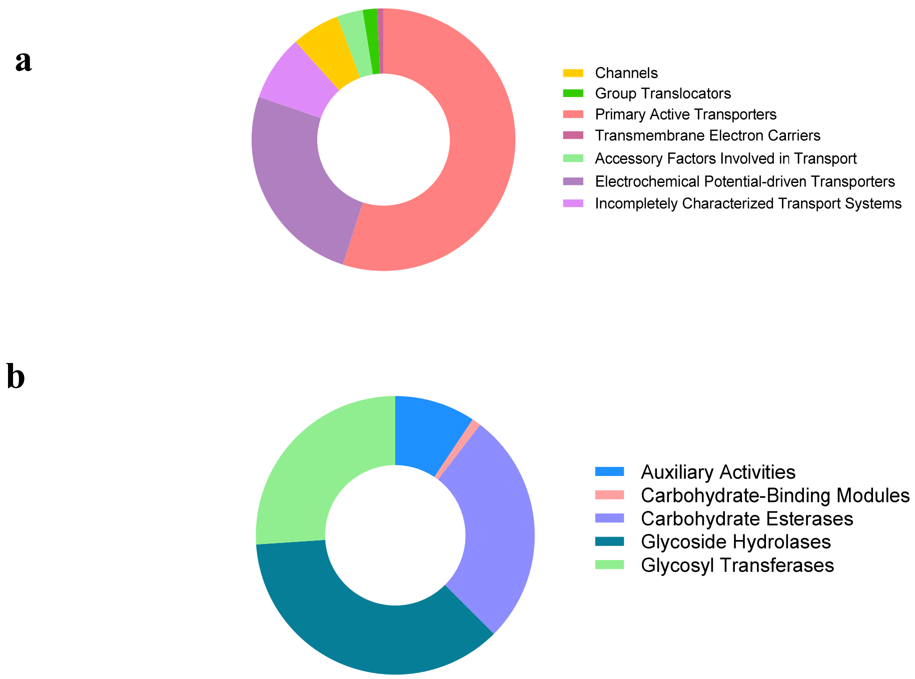

Figure S3. Types of CAZymes and transport proteins in *Microbacterium* sp. B1075. (a) Composition and abundance of transport proteins in *Microbacterium* sp. B1075. (b) Composition and abundance of CAZymes in *Microbacterium* sp. B1075.

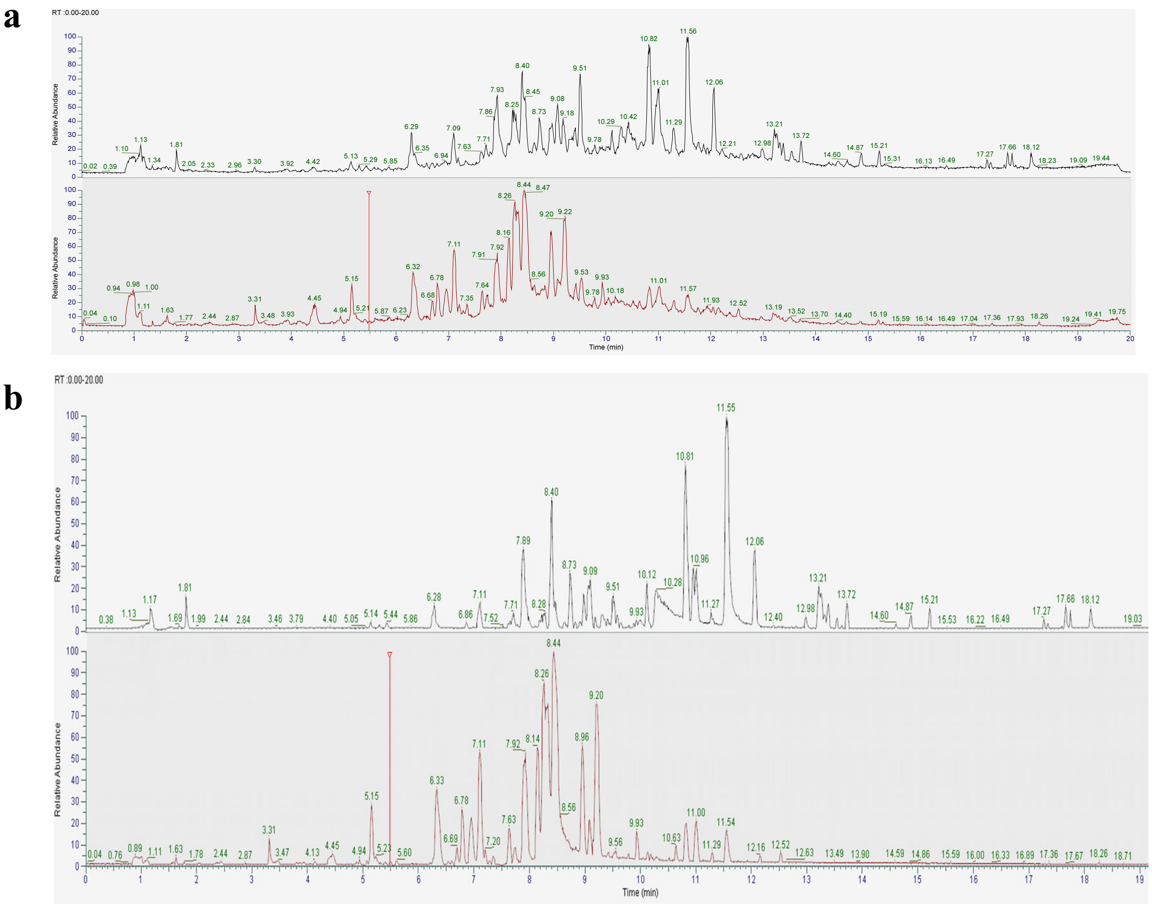

Figure S4. Secondary mass spectrometry analysis of secondary metabolites from *Microbacterium* sp. B1075, fraction Fr-2. (a) The Total Ion Chromatogram (TIC) of secondary metabolites from *Microbacterium* sp. B1075, fraction Fr-2, analyzed using UPLC-MS/MS. (b) The Base Peak Chromatogram (BPC) of secondary metabolites from *Microbacterium* sp. B1075, fraction Fr-2, analyzed using UPLC-MS/MS.

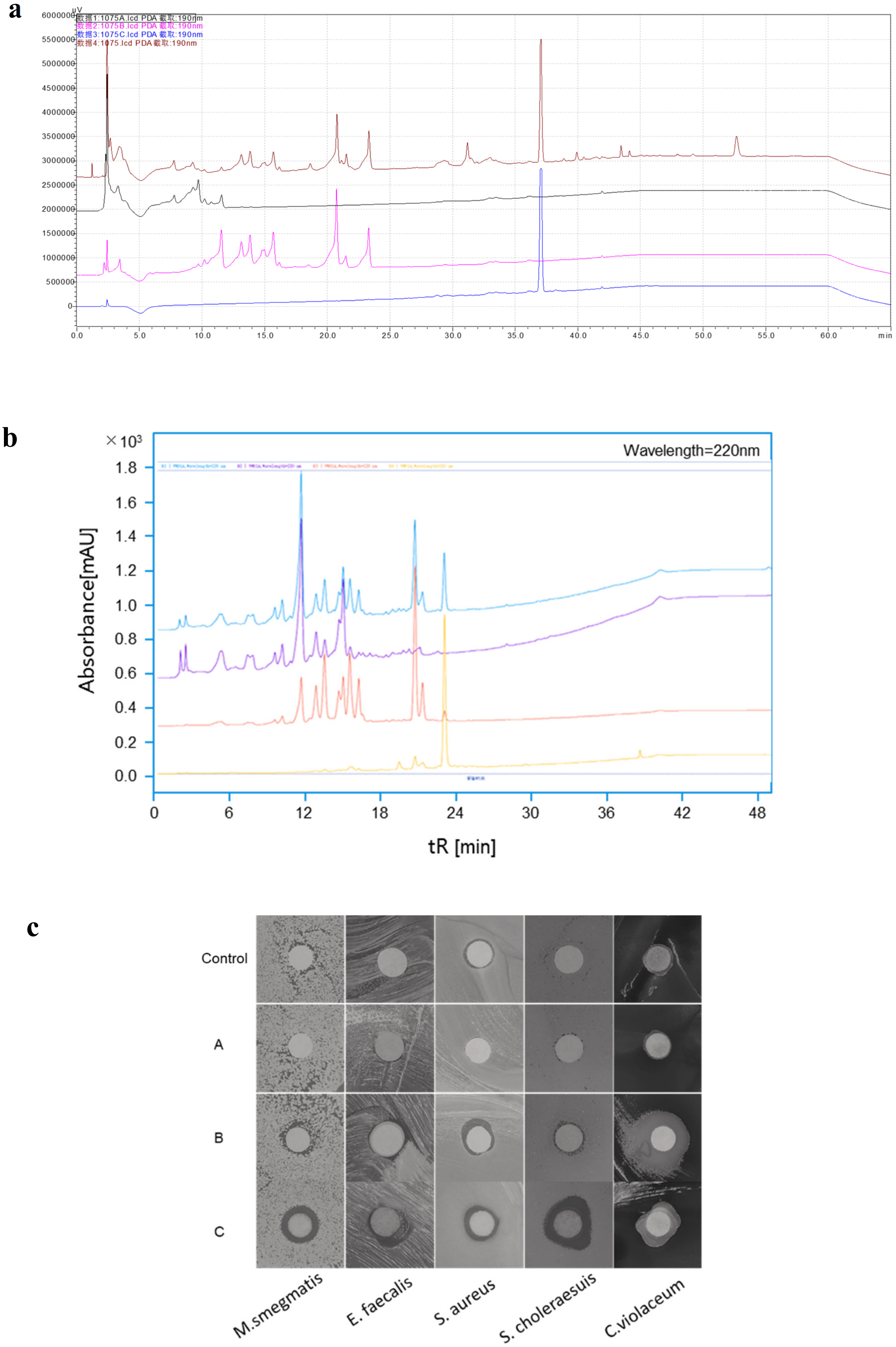
a
μV
数据1:1075A.lcd PDA 截取:190nm
数据2:1075B.lcd PDA 截取:190nm
数据3:1075C.lcd PDA 截取:190nm
数据4:1075.lcd PDA 截取:190nm
6000000
5500000
5000000
4500000
4000000
3500000
3000000
2500000
2000000
1500000
1000000
500000
0
0.0
5.0
10.0
15.0
20.0
25.0
30.0
35.0
40.0
45.0
50.0
55.0
60.0
min
b
×10³
Wavelength=220nm
Absorbance[mAU]
1.8
1.6
1.4
1.2
1.0
0.8
0.6
0.4
0.2
0.0
0
6
12
18
24
30
36
42
48
tR [min]
c
Control
A
B
C
M.smegmatis
E. faecalis
S. aureus
S. choleraesuis
C.violaceum

Figure S5. Purification and Antibacterial Activity of Secondary Metabolites from *Microbacterium* sp. B1075. (a) The brown curve represents the crude extract full segment chromatogram (1.3579 g). The black curve represents the partial chromatogram of crude extract Fr-1 (0.3318 g). The purple curve represents the partial chromatogram of crude extract Fr-2 (0.2078 g). The blue curve represents the partial chromatogram of crude extract Fr-3 (0.0564 g). The displayed data was collected at a wavelength of 190 nm using a PDA detector. (b) The blue curve represents the chromatogram of Fr-2.1, totaling 0.1220 g. The purple curve represents the chromatogram of crude extract Fr-2.2, totaling 0.0252g. The red curve represents the chromatogram of crude extract Fr-2.3, totaling 0.0594 g. The yellow curve represents the chromatogram of crude extract Fr-2.4, totaling 0.0142 g. The displayed data was collected at a wavelength of 220 nm using a VWD detector. (c) The antibacterial activity of secondary metabolites Fr-1 to Fr-3 from *Microbacterium* sp. B1075 corresponds to A, B, and C, respectively; Control represents 'control' group (methanol).

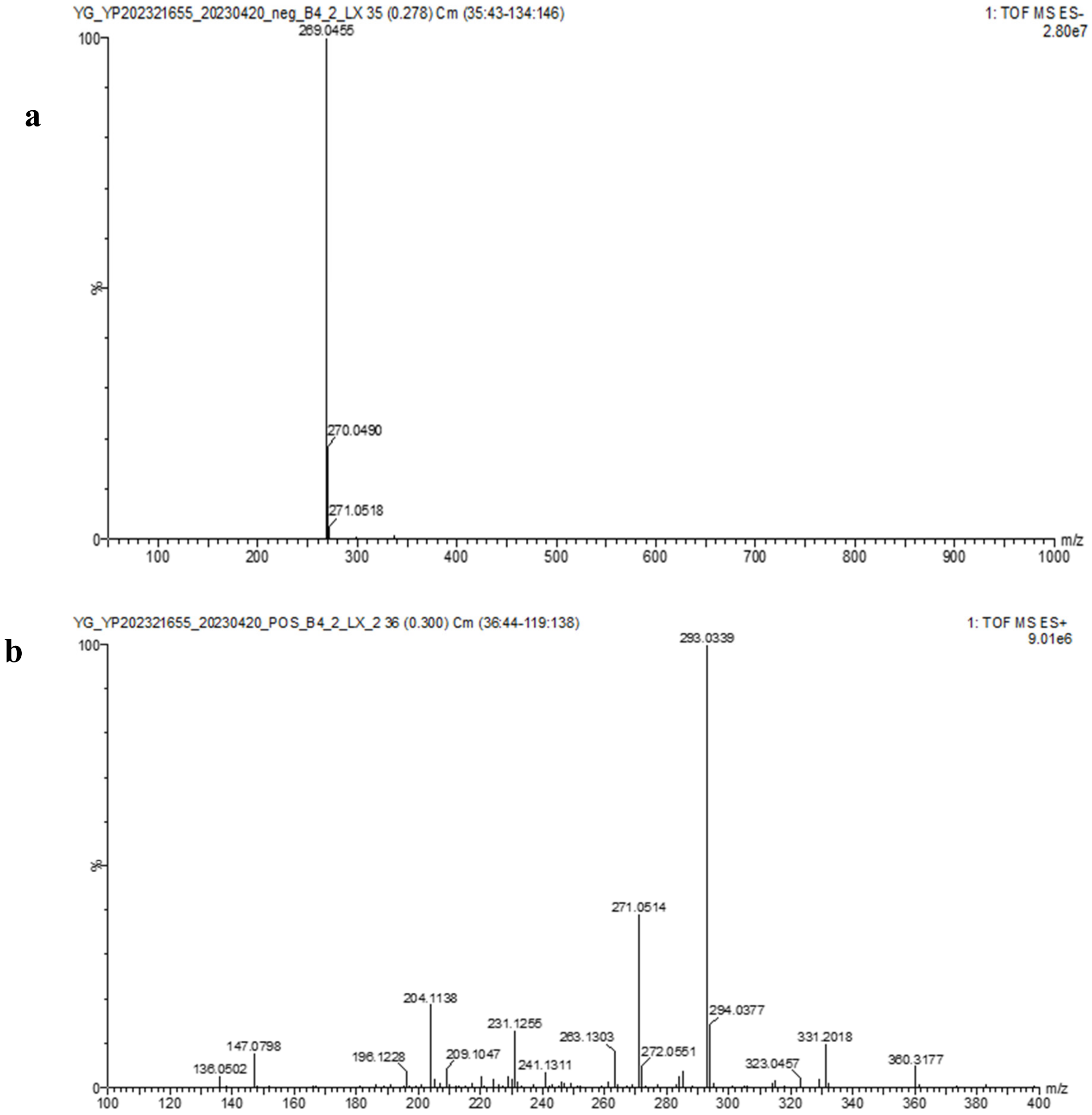

Figure S6. TOF-MS spectrum of genistein. (a) Mass spectrum of genistein in negative ion mode, m/z 50-1000. (b) Mass spectrum of genistein in positive ion mode, m/z 50-1000.

a

b

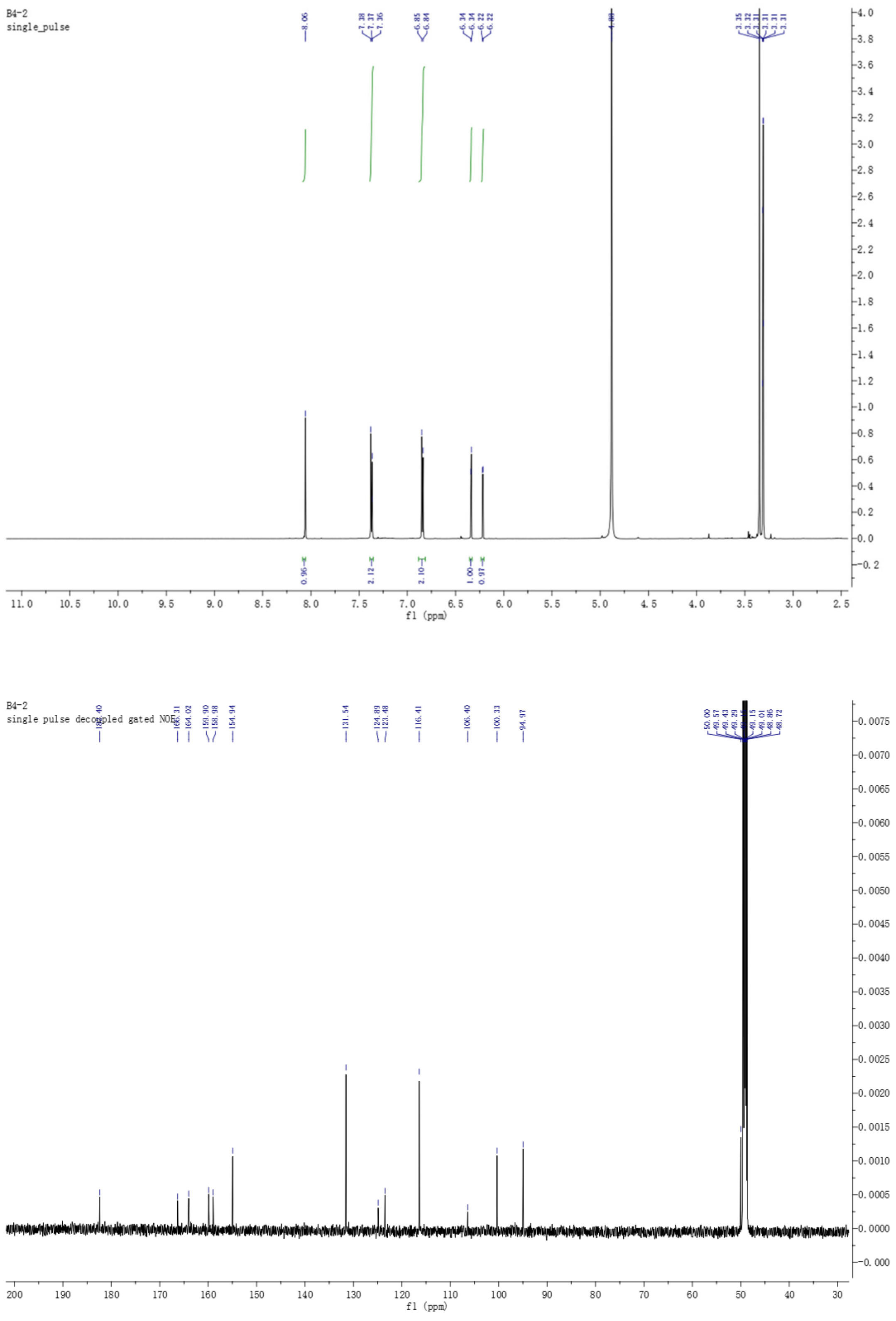

Figure S7. Nuclear magnetic resonance spectroscopy of genistein. (a) ^{1}H-NMR spectrum of genistein in CD_3OD. (b) 13C NMR spectrum of genistein in CD_3OD.

a

b

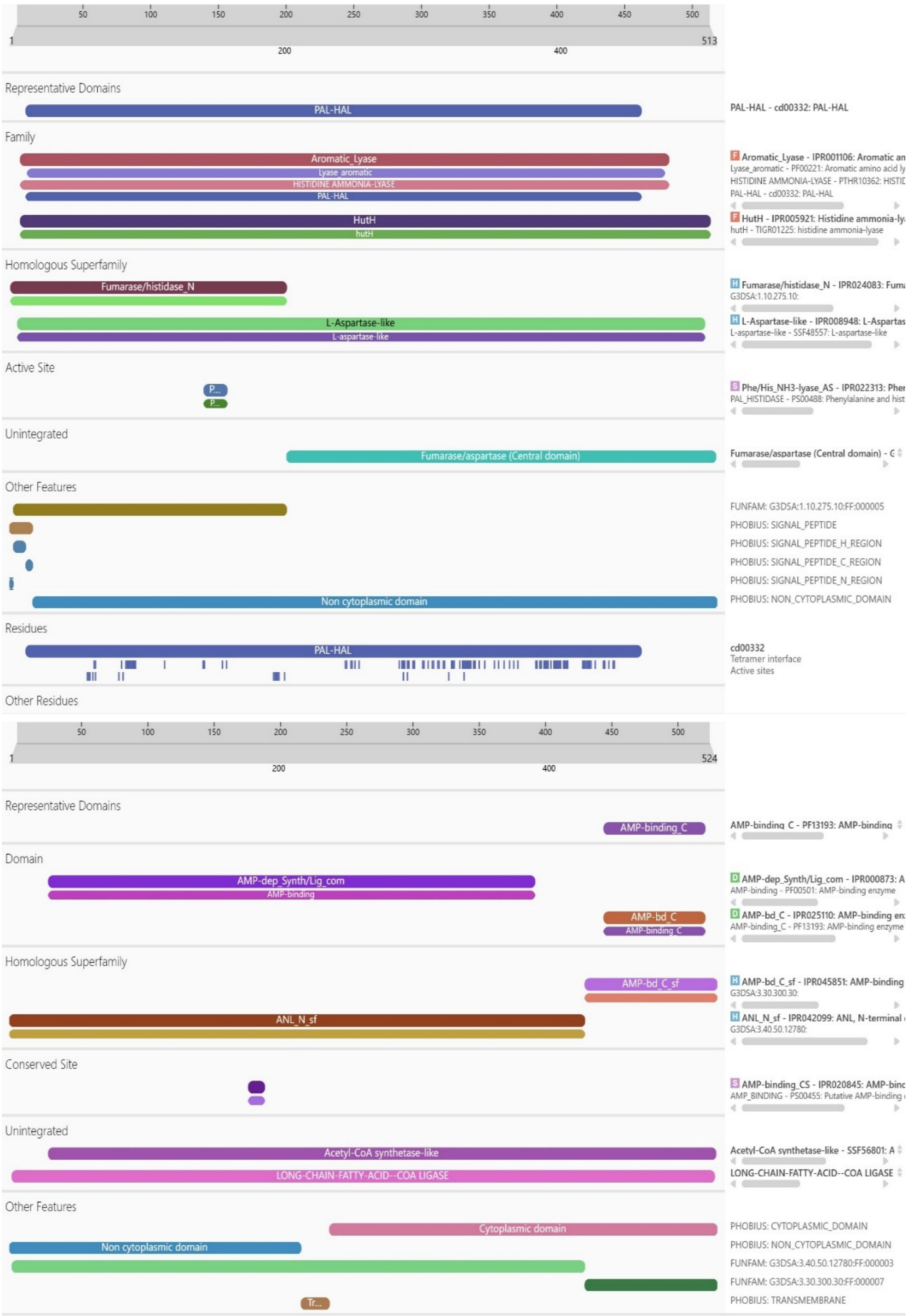

50 100 150 200 250 300 350 400 450 500
1 513
200 400
Representative Domains
PAL-HAL
PAL-HAL - cd00332: PAL-HAL
Family
Aromatic_Lyase
Lyase_aromatic
HISTIDINE AMMONIA-LYASE
PAL-HAL
HutH
hutH
Homologous Superfamily
Fumarase/histidase_N
L-Aspartase-like
L-aspartase-like
Active Site
Unintegrated
Fumarase/aspartase (Central domain)
Other Features
Non cytoplasmic domain
FUNFAM: G3DSA:1.10.275.10:FF:000005
PHOBIUS: SIGNAL_PEPTIDE
PHOBIUS: SIGNAL_PEPTIDE_H_REGION
PHOBIUS: SIGNAL_PEPTIDE_C_REGION
PHOBIUS: SIGNAL_PEPTIDE_N_REGION
PHOBIUS: NON_CYTOPLASMIC_DOMAIN
Residues
PAL-HAL
cd00332
Tetramer interface
Active sites
Other Residues
50 100 150 200 250 300 350 400 450 500
1 524
200 400
Representative Domains
AMP-binding_C
Domain
AMP-dep_Synth/Lig_com
AMP-binding
AMP-bd_C
AMP-binding_C
Homologous Superfamily
AMP-bd_C_sf
ANL_N_sf
Conserved Site
Unintegrated
Acetyl-CoA synthetase-like
LONG-CHAIN-FATTY-ACID--COA LIGASE
Other Features
Cytoplasmic domain
Non cytoplasmic domain
PHOBIUS: CYTOPLASMIC_DOMAIN
PHOBIUS: NON_CYTOPLASMIC_DOMAIN
FUNFAM: G3DSA:3.40.50.12780:FF:000003
FUNFAM: G3DSA:3.30.300.30:FF:000007
PHOBIUS: TRANSMEMBRANE

TAL

4CL

c

d

e

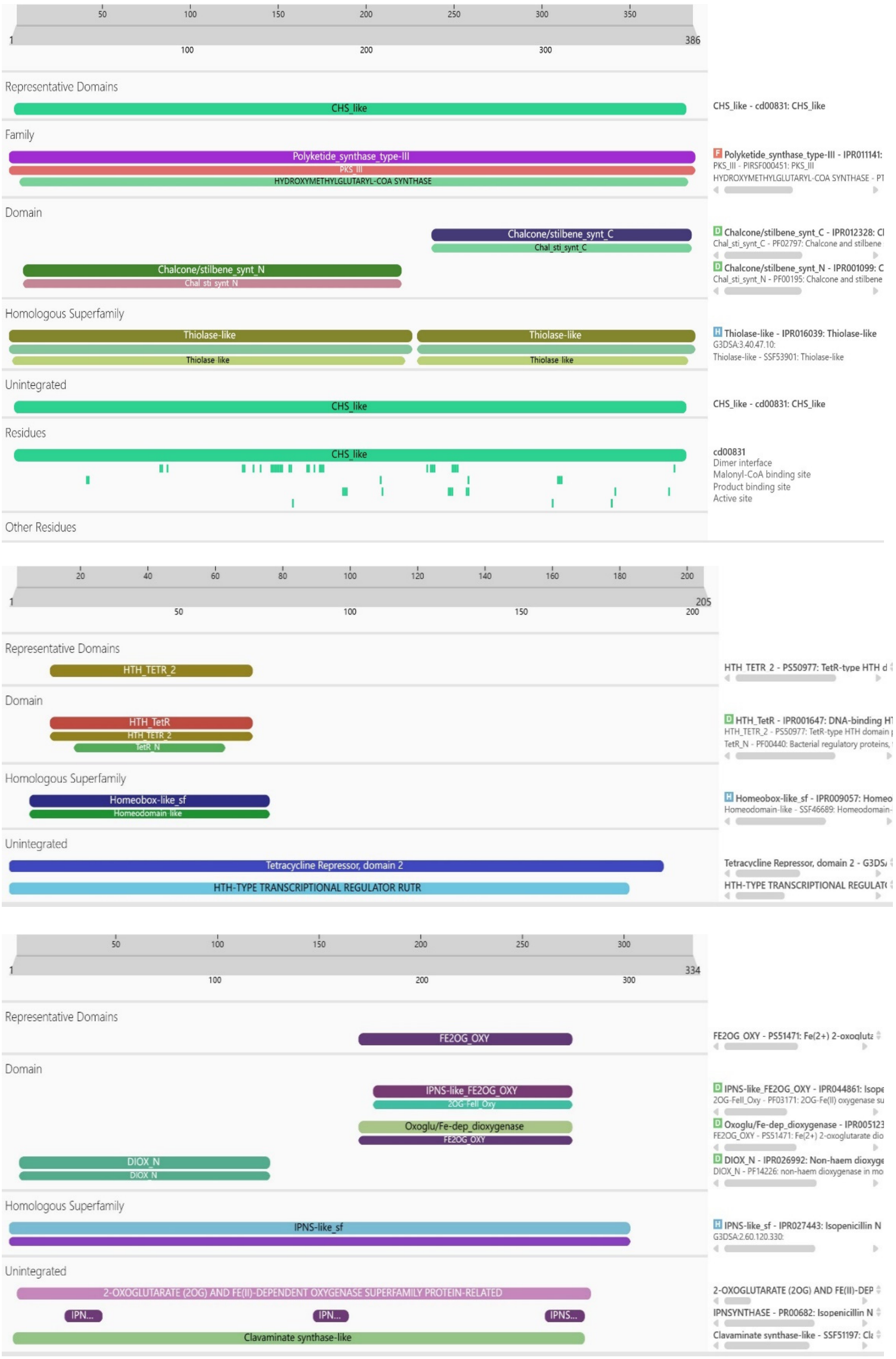

CHS

CHI

FNS

Figure S8. Predicting the structural domains of enzymes involved in flavonoid biosynthesis in *Microbacterium* sp. B1075 using InterPro**.** (a) The protein domain diagram predicted by InterPro for **tyrosine ammonia-lyase (TAL)** (PWF71_01175, WEF21307) in strain B1075. (b) The protein domain diagram predicted by InterPro for **4-Coumaroyl-CoA ligase (4CL)** (PWF71_13600, WEF20308) in strain B1075. (c) The protein domain diagram predicted by InterPro for **chalcone synthase (CHS)** (PWF71_06300, WEF22284) in strain B1075. (d) The protein domain diagram

predicted by InterPro for **chalcone isomerase (CHI)** (PWF71_14570, WEF20497) in strain B1075. (e) The protein domain diagram predicted by InterPro for **flavone synthase (FNS)** (PWF71_09720, WEF19579) in strain B1075.

Table S1. Identification of 5 purified bacteria isolated from different depths in Mariana Trench based on 16S rRNA gene sequence analyses.

Strains	Depth (m)	Top-hit type strain	Similarity (%)
B1075	5900	*Microbacterium algeriense* G1^{T}	99.78%
B755	8200	*Microbacterium binotii* CIP 101303^{T}	99.49%
B423	5900	*Microbacterium algeriense* G1^{T}	98.92%
B481	6000	*Microbacterium shaanxiense* CCNWSP60^{T}	98.92%
B419	5900	*Microbacterium maritypicum* DSM 12512^{T}	99.29%

Table S2. The secondary metabolites of *Microbacterium* sp. B1075, fraction Fr-2, were identified to contain 25 flavonoid compounds through secondary mass spectrometry data analysis in the GNPS database.

SpectrumID	Compound_Name	SpecM/Z	LibM/Z	MQScore	Adduct	Precursor_M/Z	M/ZErrorPPM	RT_Query
1	"2,2',4'-Trihydroxychalcone	256.119	257.081	0.876396	M+H	257.081	3742.03	485.613
2	2',4-Dihydroxychalcone	240.066	241.086	0.881136	[M+H]+	241.086	4230.87	783.118
3	"2',4'-Dihydroxyflavone	254.129	255.065	0.875818	M+H	255.065	3669.67	676.104
4	2'-Hydroxy-a-naphthoflavone	256.981	311.068	0.825313	M+Na	311.068	376.138	808.954
5	2'-Methoxyformonetin	256.981	297.161	0.820288	M-H	297.161	282.725	736.238
6	"3',4',7-Trihydroxyisoflavone	256.981	271.06	0.909836	M+H	271.06	309.949	587.258
7	"4',5,7-Trimethoxyflavone	256.981	313.107	0.858162	M+H	313.107	114.914	740.138
8	4'-Hydroxychalcone	256.981	223.076	0.851306	[M-H]-	223.076	4671.02	682.56
9	"7,8,2'-Trihydroxyflavone	256.981	269.046	0.907659	M-H	269.046	66.923	403.78
10	7,8-dihydroxy-3-(4-hydroxyphenyl)-4H-chromen-4-one	256.981	271.06	0.93546	[M+H]+	271.06	132.852	553.965
11	7-Hydroxy-3-Methylflavone	252.017	253.086	0.825022	M+H	253.086	4223.86	868.134
12	7-Hydroxyflavone	256.981	237.056	0.957324	M-H	237.056	8306.03	628.858
13	Acacetin Diacetate	256.981	369.097	0.858441	[M+H]+	369.097	5331.9	760.146
14	Alpha-Naphthoflavone	256.981	273.091	0.808272	M+H	273.091	7316.3	575.219
15	Apigenin	256.981	269.046	0.830401	M-H	269.046	7151.12	645.627
16	Baicalein	256.981	269.046	0.870646	M-H	269.046	3962.18	513.735
17	Cabreuvin	256.981	313.107	0.892874	M+H	313.107	6320.54	640.715
18	Daidzein	254.221	255.4	0.962193	[M+H]+	255.4	4616.29	648.205
19	Biochanin A	256.981	283.061	0.83703	[M-H]-	283.061	3543.37	632.178
20	Fisetin	256.981	287.055	0.810564	M+H	287.055	6894.16	483.046

21	Genistein	256.981	269.045	0.921894	M-H	269.045	3975.46	586.159
22	Quercetin	256.981	303.05	0.874088	M+H	303.05	6533.61	719.778
23	Isoliquiritigenin	255.799	255.066	0.8152	[M-H]-	255.066	2873.77	616.637
24	Kaempferol	256.981	285.04	0.842348	M-H	285.04	6946.43	701.287
25	Tricetin	256.981	303.05	0.861814	[M+H]+	303.05	603.907	1049.06

Table S3. Alignment results of the enzyme genes related to genistein biosynthesis in strain B1075.

Enzyme	Gene ID of strain B1075	Protein sequence identifier for strain B1075	Aligned protein sequence identifier	Similarity (%)	E-value
TAL	PWF71_01175	WEF21307	VEH25920	72%	0
4CL	PWF71_13600	WEF20308	AHE41426	42%	8.22e-09
CHS	PWF71_06300	WEF22284	GAT73531	82%	0
CHI	PWF71_14570	WEF20497	WP_187272318	29%	8.72e-09
FNS	PWF71_09720	WEF19579	QCP71067	32%	1.31e-36

Table S4. Primers required for the experiment.

**ID**	5’-3’
27F	AGAGTTTGATCATGGCTCAG
1492R	GGTTACCTTGTTACGACTT
q16SF	TCCTGGTGTAGCGGTGGAATGC
q16SR	GCGTTAGCTGCGTCACGGAATC
qCHSF1	CCGTTCTCGTCCAGGATCAGGT
qCHSR1	CCTCTCGGGCGTAGAAGTCGTT

Table S5. Formulation and pH Data of Media.

Medium	Formulation	pH
SOB	1 L distilled water, 2.0%Tryptone, 0.5%Yeast Extract, 10mM Sodium chloride, 2.5mM Potassium Chloride, 10mM magnesium chloride, 10mM Magnesium Sulfate	Natural pH
Half-strength marine agar 2216	500 mL seawater, 500 mL distilled water, 5 g peptone, 1 g yeast extract, 0.1 g FePO4 (added for screening bacteria, not for bacterial cultivation), 15 g agar.	7.6
TSB	1 L distilled water, 30 g Tryptone, 3 g yeast extract, and 15 g of agar.	7.1
LB	1 L distilled water, 10 g tryptone, 5 g yeast extract, 10 g of NaCl	7.4

16S rRNA gene sequences

> *Microbacterium maritypicum* B419

AAGGCTTTGGCGCTGCTTACACATGCAGTCGAACGGTGAACACGGAGCTTGCTCTGTG
GGATCAGTGGCGAACGGGTGAGTAACACGTGAGCAACCTGCCCCTGACTCTGGGATA
AGCGCTGGAAACGGCGTCTAATACTGGATATGTGACGTGACCGCATGGTCTGCGTCTG
GAAAGAATTTCGGTTGGGGATGGGCTCGCGGCCTATCAGCTTGTTGGTGAGGTAATGG
CTCACCAAGGCGTCGACGGGTAGCCGGCCTGAGAGGGTGACCGGCCACACTGGGACT
GAGACACGGCCCAGACTCCTACGGGAGGCAGCAGTGGGGAATATTGCACAATGGGCG
CAAGCCTGATGCAGCAACGCCGCGTGAGGGACGACGGCCTTCGGGTTGTAAACCTCTT
TTAGCAGGGAAGAAGCGAAAGTGACGGTACCTGCAGAAAAAGCGCCGGCTAACTACG
TGCCAGCAGCCGCGGTAATACGTAGGGCGCAAGCGTTATCCGGAATTATTGGGCGTAA
AGAGCTCGTAGGCGGTTTGTCGCGTCTGCTGTGAAATCCGGAGGCTCAACYYCSGGCC
TGCAGTGGGTACGGGCAGACTAGAGTGCGGTAGGGGAGATTGGAATTCCTGGTGTAGC
GGTGGAATGCGCAGATATCAGGAGGAACACCGATGGCGAAGGCAGATCTCTGGGCCG
TAACTGACGCTGAGGAGCGAAAGGGTGGGGAGCAAACAGGCTTAGATACCCTGGTAG
TCCACCCCGTAAACGTTGGGAACTAGTTGTGGGGTCCATTCCACGGATTCCGTGACGC
AGCTAACGCATTAAGTTCCCCGCCTGGGGAGTACGGCCGCAAGGCTAAAACTCAAAG
GAATTGACGGGGACCCGCACAAGCGGCGGAGCATGCGGATTAATTCGATGCAACGCG
AAGAACCTTACCAAGGCTTGACATATACGAGAACGGGCCAGAAATGGTCAACTCTTTG
GACACTCGTAAACAGKKKGTGCATGGTTGTCGTCAGCTCGTGTCGTGAGATGTTGGGT
TAAGTCCCGCAACGAGCGCAACCCTCGTTCTATGTTGCCAGCACGTAATGGTGGGAAC
TCATGGGATACTGCCGGGGTCAACTCGGAGGAAGGTGGGGATGACGTCAAATCATCAT
GCCCCTTATGTCTTGGGCTTCACGCATGCTACAATGGCCGGTACAAAGGGCTGCAATAC
CGCGAGGTGGAGCGAATCCCAAAAAGCCGGTCCCAGTTCGGATTGAGGTCTGCAACT
CGACCTCATGAAGTCGGAGTCGCTAGTAATCGCAGATCAGCAACGCTGCGGTGAATAC
GTTCCCGGGTCTTGTACACACCGCCCGTCAAGTCATGAAAGTCGGTAACACCTGAAGC
CGGTGGCCTAACCCTTGTGGAGGAGCCGTCGAAGGTGATTCATCCCC

> *Microbacterium shaanxiense* B481

GAGGCTTTGCGCTGCTACCATGCAGTCGAACGGTGAAGCCCCGCTTGCGGGGTGGATC
AGTGGCGAACGGGTGAGTAACACGTGAGCAACCTGCCCCTGACTCTGGGATAAGCGC
TGGAAACGGCGTCTAATACTGGATACGAGACGTGATCGCATGGTCAACGTTTGGAAAG
ATTTTTCGGTTGGGGATGGGCTCGCGGCCTATCAGCTTGTTGGTGAGGTAATGGCTCAC
CAAGGCGTCGACGGGTAGCCGGCCTGAGAGGGTGACCGGCCACACTGGGACTGAGAC
ACGGCCCAGACTCCTACGGGAGGCAGCAGTGGGGAATATTGCACAATGGGCGCAAGC
CTGATGCAGCAACGCCGCGTGAGGGATGACGGCCTTCGGGTTGTAAACCTCTTTTAGC
AGGGAAGAAGCGAGAGTGACGGTACCTGCAGAAAAAGCACSGGCTAACTACGTGCCA
GCAGCCGCGGTAATACGTAGGGTGCAAGCGTTATCSGGAATTATTGGGCGTAAAGAGCT
CGTAGGCGGTTTGTCGCGTCTGCTGTGAAATCTGGGGGCTCAACCCCCAGCCTGCAGT
GGGTACGGGCAGACTAGAGTGCGGTAGGGGAGATTGGAATTCCTGGTGTAGCGGTGG
AATGCGCAGATATCAGGAGGAACACCGATGGCGAAGGCAGATCTCTGGGCCGTAACTG
ACGCTGAGGAGCGAAAGGGTGGGGAGCAAACAGGCTTAGATACCCTGGTAGTCCACC
CCGTAAACGTTGGGAACTAGTTGTGGGGGTCCATTCCACGGATTCCGTGACGCAGCTA
ACGCATTAAGTTCCCCGCCTGGGGAGTACGGCCGCAAGGCTAAAACTCAAAGGAATTG

ACGGGGACCCGCACAAGCGGCGGAGCATGCGGATTAATTCGATGCAACGCGAAGAAC
CTTACCAAGGCTTGACATATACGAGAACGGGCCAGAAATGGTCAACTCTTTGGACAC
TCGTAAACAGKKGGTGCATGGTTGTCGTCAGCTCGTGTCGTGAGATGTTGGGTTAAGT
CCCGCAACGAGCGCAACCCTCGTTCTATGTTGCCAGCACGTAATGGTGGGAACTCATG
GGATACTGCCGGGGTCAACTCGGAGGAAGGTGGGGATGACGTCAAATCATCATGCCCC
TTATGTCTTGGGCTTCACGCATGCTACAATGGCCGGTACAATGGGCTGCAATACCGTGA
GGTGGAGCGAATCCCAAAAAGCCGGTCCCAGTTCGGATTGAGGTCTGCAACTCGACC
TCATGAAGTCGGAGTCGCTAGTAATCGCAGATCAGCAACGCTGCGGTGAATACGTTCC
CGGGTCTTGTACACACCGCCCGTCAAGTCATGAAAGTCGGTAACACCTGAAGCCGGTG
GCCTAACCCCTTGTGGGAGGAGCTTCGAAGGTGATCATTCC

>*Microbacterium keratanolyticum* B423
AATGCATGCGCTGCTACACATGCAGTCGACGGTGAACACGGAGCTTGCTCTGTGGGAT
CAGTGGCGAACGGGTGAGTAACACGTGAGCAACCTGCCCCTGACTCTGGGATAAGCG
CTGGAAACGGCGTCTAATACTGGATATGTGACGTGACCGCATGGTCTGCGTCTGGAAA
GAATTTCGGTTGGGGATGGGCTCGCGGCCTATCAGCTTGTTGGTGAGGTAATGGCTCAC
CAAGGCGTCGACGGGTAGCCGGCCTGAGAGGGTGACCGGCCACACTGGGACTGAGAC
ACGGCCCAGACTCCTACGGGAGGCAGCAGTGGGGAATATTGCACAATGGGCGCAAGC
CTGATGCAGCAACGCCGCGTGAGGGACGACGGCCTTCGGGTTGTAAACCTCTTTTAGC
AGGGAAGAAGCGAAAGTGACGGTACCTGCAGAAAAAGCGCCGGGCTAACTACGTGCC
AGCAGCCGCGGTAATACGTAGGGCGCAAGCGTTATCCGGGAATTATTGGGCGTAAAGA
GCTCGTAGGCGGTTTGTCGCGTCTGCTGTGAAATCCGGGAGGCTCAACCTCCGGCCTG
CAGTGGGTACGGGCAGACTAGAGTGCGGTAGGGGAGATTGGAATTCCTGGTGTAGCG
GTGGAATGCGCAGATATCAGGAGGAACACCGATGGCGAAGGCAGATCTCTGGGCCGTA
ACTGACGCTGAGGAGCGAAAGGGTGGGGAGCAAACAGGCTTAGATACCCTGGTAGTC
CACCCCGTAAACGTTGGGAACTAGTTGTGGGGTCCATTCCACGGATTCCGTGACGCAG
CTAACGCATTAAGTTCCCCGCCTGGGGAGTACGGCCGCAAGGCTAAAACTCAAAGGA
ATTGACGGGGACCCGCACAAGCGGCGGAGCATGCGGATTAATTCGATGCAACGCGAA
GAACCTTACCAAGGCTTGACATATACGAGAACGGGCCAGAAATGGTCAACTCTTTGGA
CACTCGTAAACAGGTGGTGCATGGTTGTCGTCAGCTCGTGTCGTGAGATGTTGGGTTA
AGTCCCGCAACGAGCGCAACCCTCGTTCTATGTTGCCAGCACGTAATGGTGGGAACTC
ATGGGATACTGCCGGGGTCAACTCGGAGGAAGGTGGGGATGACGTCAAATCATCATGC
CCCTTATGTCTTGGGCTTCACGCATGCTACAATGGCCGGTACAAAGGGCTGCAATACCG
CGAGGTGGAGCGAATCCCAAAAAGCCGGTCCCAGTTCGGATTGAGGTCTGCAACTCG
ACCTCATGAAGTCGGAGTCGCTAGTAATCGCAGATCAGCAACGCTGCGGTGAATACGT
TCCCGGGTCTTGTACACACCGCCCGTCAAGTCATGAAAGTCGGTAACACCTGAAGCCG
GTGGCCTAACCCTTGTGGAGGAGCCGTCGAAGGTGATCATCCATT

>*Microbacterium binotii* B755
AATGCATTGCGGCTGCTTACCATGCAGTCGAACGGTGAAGCAGAGCTTGCTCTGTGGA
TCAGTGGCGAACGGGTGAGTAACACGTGAGCAACCTGCCCTGGACTCTGGGATAAGC
GCTGGAAACGGCGTCTAATACTGGATACGAGACGTGGCCGCATGGTCAACGTTTGGAA
AGATTTTTGGTTCAGGATGGGCTCGCGGCCTATCAGCTTGTTGGTGAGGTAATGGCTC
ACCAAGGCGTCGACGGGTAGCCGGCCTGAGAGGGTGACCGGCCACACTGGGACTGAG

ACACGGCCCAGACTCCTACGGGAGGCAGCAGTGGGGAATATTGCACAATGGGCGAAA
GCCTGATGCAGCAACGCCGCGTGAGGGATGACGGCCTTCGGGTTGTAAACCTCTTTA
GCAAGGAAGRAGCGAAAGTGACGGTACTTGCAGAAAAARSSSCCGGGCTAACTACGT
GCCAGCAGCCGCGGTAATACGTAGGGCGCAAGCGTTATCCGGAATTATTGGGCGTAAA
GAGCTCGTAGGCGGTTTGTCGCGTCTGCTGTGAAAACTGGAGGCTCAACCTCCAGCCT
GCAGTGGGTACGGGCAGACTAGAGTGCGGTAGGGGAGATTGGAATTCCTGGTGTAGC
GGTGGAATGCGCAGATATCAGGAGGAACACCGATGGCGAAGGCAGATCTCTGGGCCG
TAACTGACGCTGAGGAGCGAAAGGGTGGGGAGCAAACAGGCTTAGATACCCTGGTAG
TCCACCCCGTAAACGTTGGGAACTAGTTGTGGGGACCATTCCACGGTTTCCGTGACGC
AGCTAACGCATTAAGTTCCCCGCCTGGGGAGTACGGCCGCAAGGCTAAAACTCAAAG
GAATTGACGGGGACCCGCACAAGCGGCGGAGCATGCGGATTAATTCGATGCAACGCG
AAGAACCTTACCAAGGCTTGACATATACGAGAACGCTGCAGAAATGTAGAACTCTTTG
GACACTCGTATACAGKKGGTGCATGGTTGTCGTCAGCTCGTGTCGTGAGATGTTGGGT
TAAGTCCCGCAACGAGCGCAACCCTCGTTCTATGTTGCCAGCACGTAATGGTGGGAAC
TCATGGGATACTGCCGGGGTCAACTCGGAGGAAGGTGGGGATGACGTCAAATCATCAT
GCCCCTTATGTCTTGGGCTTCACGCATGCTACAATGGCCGGTACAAAGGGCTGCAATAC
CGTGAGGTGGAGCGAATCCCAAAAAGCCGGTCCCAGTTCGGATTGAGGTCTGCAACT
CGACCTCATGAAGTCGGAGTCGCTAGTAATCGCAGATCAGCAACGCTGCGGTGAATAC
GTTCCCGGGTCTTGTACACACCGCCCGTCAAGTCATGAAAGTCGGTAACACCTGAAGC
CGGTGGCCTAACCCTTGTGGAGGAGCCGTCGAAGTGATCATCCC
